# QBrainNet: harnessing enhanced quantum intelligence for advanced brain stroke prediction from medical imaging

**DOI:** 10.3389/fmed.2025.1677234

**Published:** 2025-10-23

**Authors:** M. Priyadharshini, V. Murugesh, T. R. Mahesh, Eid Albalawi, Oumaima Saidani, Ali Algarni

**Affiliations:** ^1^Department of Computer Science & Engineering, Faculty of Science and Technology (IcfaiTech), ICFAI Foundation for Higher Education, Hyderabad, India; ^2^School of Computer Science, Coventry University Kazakhstan, Astana, Kazakhstan; ^3^Department of Computer Science and Engineering, Faculty of Engineering and Technology, JAIN (Deemed-to-be University), Bengaluru, India; ^4^Department of Computer Science, College of Computer Science and Information Technology, King Faisal University, Al Ahsa, Saudi Arabia; ^5^Department of Information Systems, College of Computer and Information Sciences, Princess Nourah bint Abdulrahman University, Riyadh, Saudi Arabia; ^6^Department of Informatics and Computer Systems, College of Computer Science, King Khalid University, Abha, Asir, Saudi Arabia; ^7^Center for Artificial Intelligence King Khalid University, Abha, Asir, Saudi Arabia

**Keywords:** brain stroke prediction, early stroke detection, medical imaging, quantum computing, quantum neural networks (QNN), quantum intelligence

## Abstract

**Introduction:**

Brain stroke is still one of the leading causes of death and long-term disability in the world. Early and correct diagnosis is therefore important for patient outcome. Although Convolution Neural Network (CNN), classical machine learning models, have achieved great progress in medical image classification, they have to face the performance saturation problem when dealing with high-dimensional and complex data such as medical images. To tackle these limitations, we propose QBrainNet, a quantum enhanced model, which is to enhance brain stroke prediction from medical imaging datasets.

**Methods:**

The model consists of Quantum Neural Networks (QNNs) applied as learning complex patterns in terms of medical images and Variational Quantum Circuits (VQCs) that will be used to optimize the classification. The feature extraction featured in the QNNs utilises quantum properties of superposition and entanglement to extract non-linear high-dimensional patterns in images related to stroke that may not be captured using classical limits. The VQCs, in turn, are applied to optimize the model performance, further allocating the boundaries of the decision and enhancing the model performance in terms of accuracy by optimizing the quantum gates and operators used during the work. QBrainNet utilizes the combination of such quantum properties as entanglement and superposition to represent more complicated non-linear patterns in stroke-specific images in a better manner than a classical application does.

**Results:**

This paper proposes a hybrid classical-quantum scheme: preprocessing classically, and learning quantum-enhanced. Quantum gates and operators are used when performing the quantum phase to optimize decision boundaries, achieving vastly enhanced prediction accuracy and efficiency performance. Experimental results indicate that QBrainNet has a better accuracy (96%) and AUC-PR (0.97) than the classical models like CNN, SVM, and Random Forest, proving the superior performance of QBrainNet in stroke detection.

**Discussion:**

The inference time is shorter, so the model can be used as a real-time clinical application. This article points to the possibilities quantum computing can have in revolutionizing medical diagnostics, especially stroke prediction.

## 1 Introduction

Stroke constitutes one of the significant causes of death and permanent disability in the world, with about 15 million individuals having a stroke per year according to the WHO (1). Early diagnosis and prompt treatment are essential in enhancing survival and minimizing long-term disability. Nevertheless, clinical condition diagnosis, where time is of the essence, will still be a challenge to correctly predict because of the complexity and subtlety of patterns in medical imaging data, particularly in the early stages (2, 3). Interpretation of CT and MRI scans used widely to detect stroke is subject to human error, inconsistency, and variability between practitioners, and it may lead to delay in diagnosis and impact treatment outcomes (4).

Recently, the methods based on machine learning (ML), particularly Convolutional Neural Networks (CNNs), have been actively applied to the medical image analysis, and stroke detection has been successful with the CT or MRI scans. The CNNs have been shown to work exceptionally well when processing medical imagery and extracting features that classify the image as stroke-related quickly, consistently, and accurately, compared to the more conventional methods (5, 6). Although these CNNs and other classical models are effective, they are limited by high-dimensional and complex medical data. These models fail to identify delicate structures and interactions within the data, particularly when the datasets are small and/or low-contrast, as frequently happens in medical imaging of stroke patients (7, 8).

The new area of Quantum Machine Learning (QML) offers an optimistic answer to these difficulties. Quantum systems work with information in radically new ways compared to classical systems, allowing them to work with extensive multi-dimensional data more efficiently through superposition and entanglement. Indeed, the quantum properties allow quantum computers to solve some problems efficiently in computation, where classical computers do not; the quantum potential advantage has indeed been observed in applications such as medical image analysis (9, 10). Quantum Neural Networks (QNNs) and Variational Quantum Circuits (VQCs) can specifically be used to provide an advantage in the classical world in specific tasks by finding complex patterns and relationships in data and using these patterns and traits in a non-linear fashion (11, 12).

This paper presents QBrainNet, a classical-quantum model that aims to enhance medical imaging stroke prediction. The classical element of the QBrainNet engages in feature extractions, augmenting images, and noise elimination, whereas the quantum element continuously applies QNNs and VQC networks to the learning task. QBrainNet, with its quantum-enhanced learning combining classical machine learning, is much faster and has a higher accuracy at identifying subtle factors in stroke-related medical images (13, 14). The quantum aspect of the model applies simulated quantum operations through Python code to optimally determine decision boundaries in the feature space. It is, therefore, more accurate in the classification than the conventional methods.

One main issue with medical image classification tasks is the small datasets. In our scenario, we only have 3,800 images, which can easily result in overfitting. However, the problem can be overcome the way QBrainNet does it by using cross-validation and regularization techniques (15, 16). The quantum elements of QBrainNet are designed through Python-based quantum simulation, in which quantum gates and circuits are simulated on a classical computing device. Thus, the model is accessible and reproducible without quantum information technology hardware (17, 18).

The main strengths of the QBrainNet model in comparison with classical approaches are linked to the possibility of dealing better with high-dimensional data. CNNs and other traditional techniques are bulky programs that handle big chunks of data, particularly in the case of medical image tests. Compared to this, QBrainNet takes advantage of quantum parallelism, where quantum gates and superposition significantly decrease the degree of computation and speed of processing (19). Such a decrease in computational demands and the increase in the prediction speed result in QBrainNet being a potential candidate in clinical practice, where the speed of diagnosis may be a matter of life and death.

In recent developments, quantum computing has demonstrated great potential to improve machine learning models, particularly for high-dimensional data analysis. In this work, we simulate the quantum parts of QBrainNet using PennyLane on classical computing resources. This way, we can exploit quantum effects like superposition and entanglement for feature extraction and optimization without access to real quantum hardware. Our simulation allows us to simulate quantum circuits and perform parameter optimization in a way compatible with classical machine learning.

The present study adds to the list of research that deals with the application of quantum computing in healthcare. In particular, we show promise of quantum-enhanced models such as QBrainNet in the field of stroke prediction, namely that quantum technology can be used to enhance the performance of medical diagnostics not only in accuracy, but in efficiency as well, especially in a domain where errors can have severe consequences like stroke care (19).

## 2 Related work

Applying machine learning (ML) to medical imaging has entirely transformed the face of healthcare diagnostics in a way no one had previously imagined. More specifically, CNNs have found a wide application in deep learning to solve specific tasks in medical imaging. The application of CNNs to the interpretation of medical images has been demonstrated to be capable of detecting and classifying ailments such as cancer, pneumonia, and brain stroke, as well as segmenting organs and other body parts critical to the human body (20). Of particular interest in brain stroke detection is that CNNs and other forms of deep learning have been applied to CT image processing, MRIs, and fMRI to provide brain stroke risk assessments, but with high levels of automation. Such models are much superior in the detection of stroke lesions and the classification of ischemic strokes. By extracting hierarchical representations of image information, these models can discover useful trends that the human expert may not be able to declare easily. The approach here is a novel application of the idea behind hybrid quantum-classical neural networks (21) to predicting strokes through quantum-enhanced preprocessing.

These models, although effective, are restricted. Brain images can be complex, leading to difficulties for classical CNNs to apply to them and subtle features in the early stages of strokes. These models require substantial labeled data, computer power, and a preprocessing mechanism (22), and thus are not readily applicable to high-dimensional data. Additionally, it is computationally costly to train deep learning models wherein the high-resolution medical images are to be used; they require both heavy computing hardware and time. Original CNNs inherently lack the flexibility to extract subtly non-linear structures in the data, and such patterns are typical with medical images, as the data are noisy, heterogeneous, and may be inaccurately annotated (23). Also, this fulfills the need for more complex models that could better predict the nature of medical imaging with a complex structure (24).

To overcome these shortcomings, Quantum Machine Learning (QML) has proposed itself as an excellent solution. It is theorized that QML methods will be able to utilize the quantum superposition and quantum entanglement properties of quantum computers to both process complex information more effectively and prevent the scale explosion that occurs when using classical models. These quantum benefits may bring computational advantage, especially where data is needed in very high dimensions, such as in medical image processing (25). Quantum systems offer the prospect of investigating multiple solutions in parallel and exhibit greater capabilities of pattern recognition, which are of particular interest with complicated medical data. This will enable quantum methods, even when implemented on classical platforms using Python code, to perform better when compared with classical models in specific tasks requiring subtle non-linear relationships, e.g., when used to predict stroke (26, 27).

Healthcare and medical diagnosis are some examples in which QML has already been proven effective. For instance, Quantum Support Vector Machines (QSVM) were used to solve tasks in image classification. The results revealed that QSVMs are more effective in terms of computational efficiency than SVMs and are highly accurate in prediction (28). Moreover, QNNs, or the quantum analog of normal neural networks, have already been used in such tasks as image classification and drug discovery. Quantum-enhanced models, on the other hand, can access the power of quantum entanglement to learn intricate structures in data that are favorable over conventional models in the task of image classification (29). As some examples, the Quantum version of standard neural networks, namely Quantum Neural Networks (QNNs), have been implemented in problems like image classification and drug recognition. In the light of this understanding, QE models can leverage quantum entanglement to learn complex patterns in the data in a more efficient way than classical models, which is a key advantage in various tasks, such as image classification. Such methods are currently being utilized in this work as simulated quantum operations that, even though they do not run on actual quantum devices, act as a step in the right direction as applied to quantum-enhanced optimization.

Other quantum algorithms are likely to prove useful in healthcare, including Quantum Random Forests (QRF) and Quantum k-Nearest Neighbors (QK-NN), which have been found in many cases to require less time to train and achieve higher accuracy than their classical counterparts on high-dimensional data (30, 31). Quantum algorithms, including Quantum Random Forests (QRF) and Quantum k-Nearest Neighbors (QK-NN), have also been investigated in healthcare and on high-dimensional data. Quantum algorithms are more efficient in their training speed, and their results are found to be better when compared to classical algorithms. Such algorithms are emulated via quantum operations on a classical computer in Python and demonstrate the possibilities of the quantum-enhanced models without involving the actual physical quantum device (27).

Although applying QML to medical imaging is gaining more attention, it has not yet been explored in brain stroke prediction. Although past works have used quantum models in image segmentation, disease categorization, and other medical imaging applications, there has yet to be a quantum learning model to predict stroke occurrence using medical imagery, which is the novelty of this paper. A quickly expanding volume of literature on QML shows that one of its uses can be better optimization, image classification, and pattern recognition. Still, using QML in stroke prediction in medical imaging has yet to be explored (32). Though numerous cases of research on QML exist, there is a significant lacuna in its application in the prediction of brain stroke, which is the novelty of this work. Though quantum-enhanced models have already demonstrated their potential in optimization, image classification, and pattern-recognition problems, their use in medical imaging, in general, and stroke prediction, in particular, has not been studied extensively. This work bridges this gap through simulated quantum operations (through Python code) on classical computing resources (33).

The novelty of this research is that QBrainNet is the first application of QML in stroke prediction. The architecture can close a substantial research gap in stroke detection research as it has integrated quantum-enhanced preprocessing, feature extraction, and classification into a single framework. Classical simulations of quantum operations allow for avoiding quantum hardware, but increase the stroke prediction accuracy and reduce computing costs (34). The proposed work is the initial implementation of QML regarding stroke expectations. Quantum-based benefits to preprocessing, feature extraction, and classification strongly occur within the same framework, as all other quantum manipulations are performed through Python codes running on a classical CPU. Employing simulated quantum operations over quantum hardware indicates a big leap toward actualizing quantum-powered healthcare tools. It influences how quantum computing can be used to develop solutions to mitigate modern medicine’s challenge to the detriment of the overall healthcare industry: stroke diagnosis (35).

## 3 Methodology

This section describes the general strategy used to get to and test QBrainNet, a quantum augmented neural network that will predict the risk of stroke from brain imaging data. It contains four main parts of methodology that are dataset preparation, preprocessing and feature extraction, quantum machine learning model development and model training and evaluation. We describe each stage in detail to provide a detailed account of how the quantum techniques are integrated into the medical image analysis pipeline for increasing the accuracy of stroke prediction.

The system requirements for running the quantum operation simulations are as follows: The simulations have been run on a system that has Intel i7 processor and 16 GB RAM the Ubuntu 20.04 operating system. The quantum operations were simulated with PennyLane, version 0.18.0, a Python-based library which can build on classical computing resources to simulate quantum operations. The simulate codes were written in Python 3.8 and some additional libraries such as Numpy 1.21.0 for numerical computing, Scipy 1.7.0 for scientific computing, matplotlib 3.4.3 for visualization. The entire setup was done in a conda environment to handle everything in the appropriate way in terms of dependencies and reproducibility. This environment allowed efficient implementation of quantum simulations on classical computing resources without the need for any actual quantum hardware.

### 3.1 Dataset

The medical images included in this study were diagnosed as usual or as stroke from a dataset. The photos are taken from publicly available datasets usually used in the stroke detection area, such as CT scans and MRI images. This dataset contains high-resolution MRI brain scans of different stroke severity, early ischemia, and late-stage hemorrhage. The pictures are marked to help define which ones are routine and which have an indication of a stroke. These images are then fed through simulated quantum operations to improve feature extraction, classification, and overall predictive accuracy with Python-based quantum simulators on classical computing resources. Lastly, each image has a label, indicating whether the brain imaging is standard or if there is a stroke.

**Figure 1:**
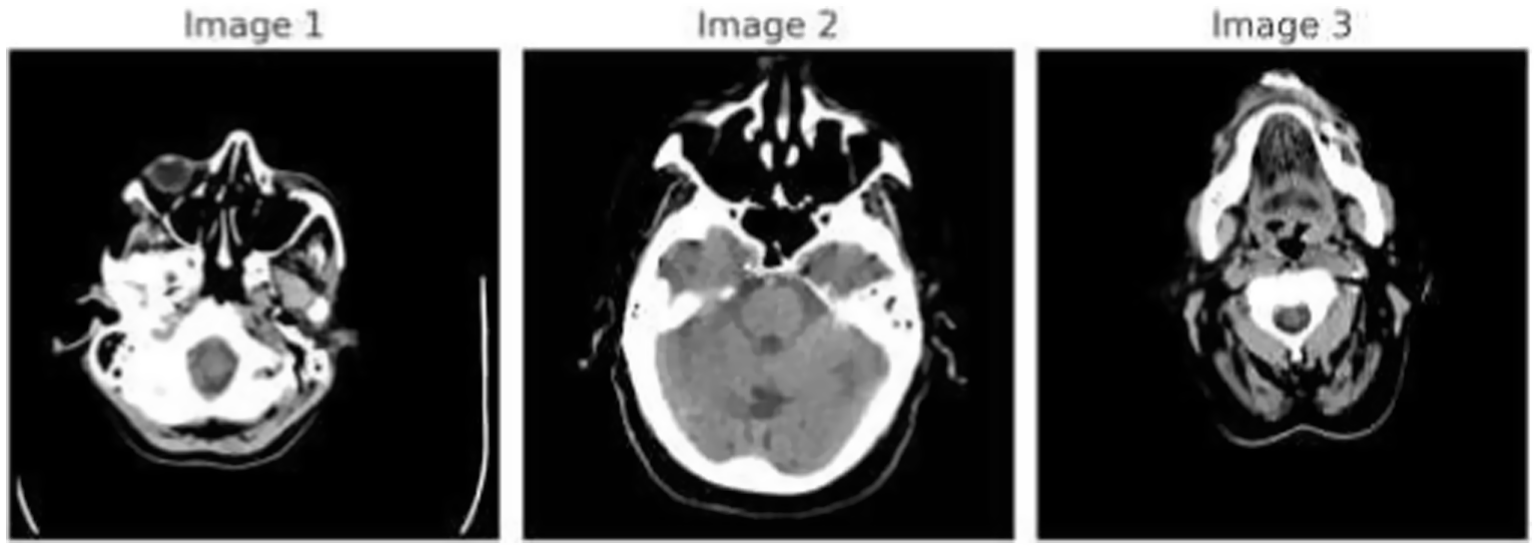
Dataset overview: raw and processed brain CT scan images.

**Figure 2:**
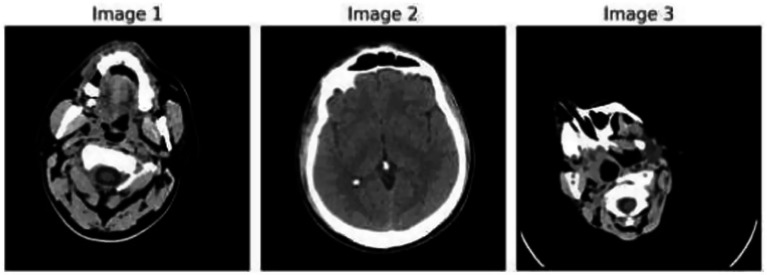
Preprocessed brain images: grayscale, equalized, and edge-detected versions.

**Figure 3:**
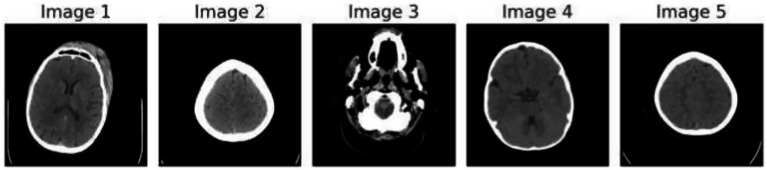
CT scan cross-sections showing brain structure and potential abnormalities.

**Figure 4:**
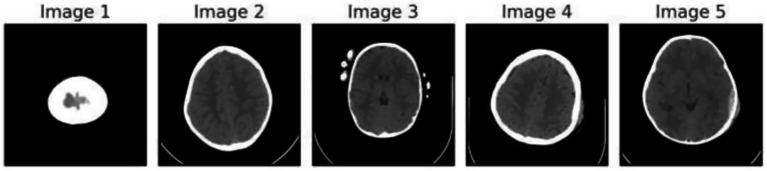
CT scan cross-sections of brain showing stroke variants.

Figure 1 demonstrates the unprocessed and processed CT scan brain scans. Raw images are initially scanned, whereas the processed ones have undergone a procedure of removing noise and normalization to facilitate analysis. Figure 2 shows grayscale, equalized, and edge-detected images of the preprocessed brain images. Gray levels eliminate color, equalization increases contrast, and edge detection emphasizes boundaries of key structures. The CT scan cross-sections shown in Figure 3 are used to obtain details about brain structure and the parts prone to abnormalities such as strokes and tumors. Figure 4 shows different CT scan cross-sections with varying types of stroke, and how ischemic and hemorrhagic strokes can be represented in the brain in a cross-section.

### 3.2 Data preprocessing

The raw medical images are preprocessed before training and evaluation to reduce inconsistency and robustness across the medical image set. Rotation, flip, and noise addition augment the dataset and make it more diverse. To resemble real data and increase the model robustness to imperfect data, these procedures simulate real-world variation, e.g., to some extent, by the slight changes in rotation or orientation of scan images, and provide noise. This can better generalize the model, especially with a small data set, as it minimizes the chances of overfitting.

The primary preprocessing steps include:

**1  Image Resizing**: Uniformity is guaranteed in the input data, as all the images in medical images may have different resolutions. They are all resized to a fixed resolution. This is an essential step so that the data maintained between multiple images is compatible with deep learning models image resizing is computed using Equation 1.

(1) $$I_{\text{resized}}=\text{Resize}(I_{\text{original}},W,h)$$

Where:

-
$I_{\text{resized}}$ - resized image.
-
$I_{\text{original}}$ - original image.
- W & h are the target width and height, respectively.

**2  Normalization**: To adjust to the different pixel intensity values represented by various medical imaging modalities, the images are scaled to the 0–1 range. This will allow the model to be adjusted only to the scale of the raw data and not be distorted by the ranges of pixel intensities normalization is computed using Equation 2.

(2) $$I_{\text{normalized}}=\frac{I_{\text{original}}}{255}$$

Where:

-
$I_{\text{normalized}}$ - normalized image.
-
$I_{\text{original}}$ - original pixel intensity.

**3  Class Imbalance Check**: Since the medical datasets usually become class imbalanced, balancing the number of samples in training and test sets within normal and stroke groups is very important. If an imbalance is discovered, methods that include over-sampling the minority observations or under-sampling the majority can be used to generate a balanced dataset. This eliminates the possibility of biasing the model toward one of the classes, which is used a lot more; hence, the model will perform well in both classes.

### 3.3 Dataset partitioning

The data is split into the training data and a testing data where 70–80 percent of the data is used in the training and 20–30 percent for testing. The training data is then trained on the model, known as QBrainNet model and the testing data is used to estimate the model’s performance on unknown data. This division will ensure the model is tested on data that it has not encountered previously during the model’s training, and will be an impartial representation of how well the model is performing.

Preprocessing of dataset, and splitting the preprocessed dataset into training and testing datasets is done. The model is trained on the training data and tested on the test data (36, 37). The training is usually done using 70–80% of the data; the remaining 20–30% is used for testing. There is a need to fold this type to make sure that the model performs well on the unseen data rather than being too optimistic regarding the performance.

#### 3.3.1 Dataset distribution

The distribution of ‘normal’ and ‘stroke’ images over training and test sets can be viewed in Table 1.

**Table 1:** Distribution of normal and stroke images in the dataset.

Class	Training set (images)	Test set (images)	Total images	% in training set	% in test set	Augmentation applied	Primary data source
Normal	1,500	500	2,000	51.70%	55.60%	Rotation, Flip, Noise	Hospital A & Public Dataset
Stroke	1,400	400	1,800	48.30%	44.40%	Contrast Stretching, Zoom	Hospital B & Research Cohort
Total	2,900	900	3,800	100%	100%	–	–

Class distribution plays a role in training the model on a balanced set of examples, which is very important for accurate stroke prediction.

#### 3.3.2 Dataset flow diagram

**Figure 5:**
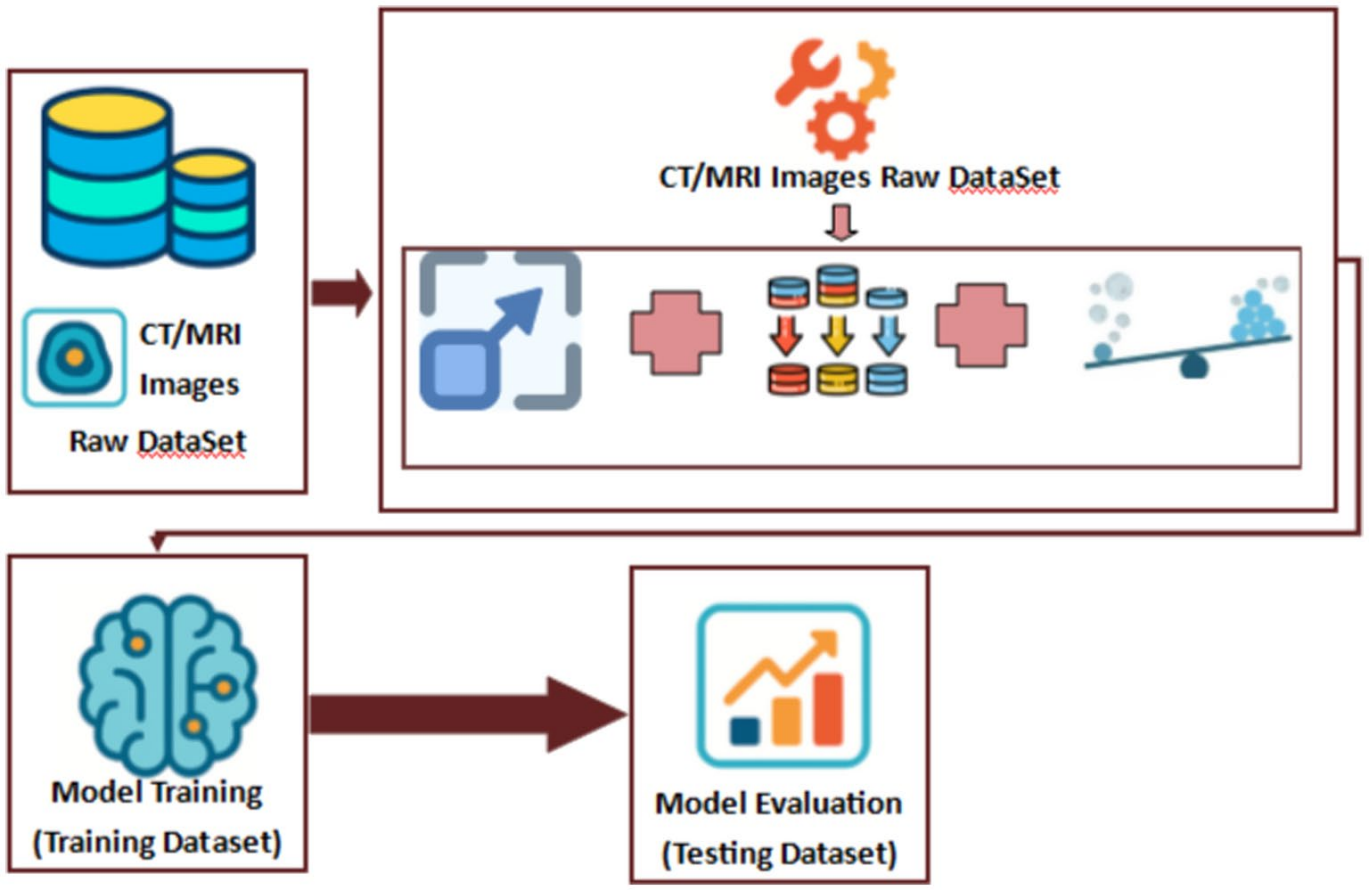
Proposed model’s dataset flow diagram.

Here, in the following Figure 5, we show the flow of the dataset in the preprocessing, training and evaluation stages:

#### 3.3.3 Class imbalance handling

To solve the problem of class imbalance of the dataset, we used some oversampling and undersampling methods during the data preprocessing phase:

**1  Oversampling**: We applied Random Oversampling to replicate samples from the minority class (either “normal” or “stroke”) to train the model on a balanced dataset. This method copies minority class samples to make the sizes of the minority and majority classes equal, eliminating the model’s bias for the majority class.

- **Stage in Pipeline**: Random Oversampling was used as one of the pipeline steps on the training set after splitting the dataset into training/validation sets. This helped ensure the model would learn from an even distribution of the two classes.

**2  Undersampling**: Since it is a class imbalance problem, we applied the Random Undersampling technique to the majority class. This method addresses the issue by randomly selecting samples from the majority class to obtain a balanced distribution between both classes. Decreasing the number of majority class samples ensures the model does not become biased toward majority class predictions.

- **Stage in Pipeline**: Minority class was oversampled, and then Random Undersampling was implemented to achieve class balance without overfitting of the minority.

**Class Imbalance Handling Pipeline**:

1. Divide the dataset into a training and validation dataset.
2. Implement Random Oversampling to the minority class in the training dataset to balance the class distribution.
3. Random Undersampling: the oversized majority class in the train data set is reduced to the size of the minority class.
4. The balanced training set is now used to train the QBrainNet model.

These techniques allow for equal representation of both classes (regular versus stroke) during model training, which is essential in healthcare applications where accurate classification of both conditions is crucial.

### 3.4 Preprocessing and feature extraction

Several classical preprocessing techniques are performed before the quantum machine learning algorithms are used to preprocess the medical images, such that the data is in a format that is as best as possible for extracting features and the model can be trained on. These techniques allow us to mitigate noise, clean, increase contrast, and standardize the stroke dataset to facilitate the networks’ detection of stroke features more easily (38).

#### 3.4.1 Image resizing

Resizing images is a crucial preprocessing step because all the images need consistent dimensions supported by deep learning models, which usually need uniform input sizes. The resizing process involves mapping the original image size $W_{\text{original}}\times h_{\text{original}}$ to a new size $w_{\mathrm{new}}\times h_{\mathrm{new}}$ . This can be mathematically represented as using Equation 3:


(3) $$I_{\text{resized}}(x,y)=I_{\text{original}}(\frac{x}{w_{\text{original}}}\cdot w_{\mathrm{new}}=\frac{y}{h_{\text{original}}}\cdot h_{\mathrm{new}})$$

Where:

-
$I_{\text{resized}}$ - resized image.
-
$I_{\text{original}}$ - original image.
-
$W_{\text{original}}$ and $h_{\text{original}}$ are the original width & height of the image.
-
$W_{\mathrm{new}}$ and $h_{\mathrm{new}}$ are the target width & height for resizing?

The bilinear interpolation method is used for resizing to preserve image details (39).

#### 3.4.2 Grayscale conversion

Grayscale conversion of the images is applied to simplify the data and decrease computational complexity while retaining stroke-related features. Grayscale images are beneficial as they decrease the number of channels (from 3 in RGB to 1), thus reducing the amount of computation and emphasizing the textural differences in the brain tissue.

The conversion from a color image $I_{\mathrm{rgb}}(x,y)$ to grayscale $I_{\text{gray}}(x,y)$ is done by averaging the weighted sum of the RGB channels, following the formula as shown in Equation 4:


(4) $$\begin{array}{l} I_{\text{gray}}(x,y)=0.2989\cdot I_{\mathrm{rgb}}^{R}(x,y)+0.5870\cdot I_{\mathrm{rgb}}^{G}(x,y)\\ +0.1140\cdot I_{\mathrm{rgb}}^{B}(x,y) \end{array}$$

Where:

$I_{\mathrm{rgb}}^{R}(x,y),I_{\mathrm{rgb}}^{G}(x,y),I_{\mathrm{rgb}}^{B}(x,y)$ - Represent the Red, Green, and Blue (RGB) color channels, respectively.

$I_{\text{gray}}(x,y)$ - resulting grayscale image.

#### 3.4.3 Histogram equalization

To enhance the contrast of the images, histogram equalization is used to redistribute the intensity levels throughout the image. Spread out across the whole range, this process helps to bring out subtle details, including early signs of stroke. Histogram equalization can be mathematically formulated as shown in Equations 5 and 6:


(5) $$\mathrm{CDF}(i)=\sum_{j=0}^{i}p(j)$$

(6) $$I_{\mathrm{eq}}(x,y)=\mathrm{CDF}(I_{\text{original}}(x,y))\cdot (L-1)$$

Where:

CDF(i) It is the cumulative distribution function of the pixel intensities.

p(j) It is the probability density function of the pixel intensities.

L Is the number of possible intensity levels (typically 256 for 8-bit images).

$I_{\mathrm{eg}}(x,y)$ It is the histogram-equalized image.

It ensures that the pixel intensity distribution is more uniform than it is, thereby improving the contrast of the image and bringing out finer details, which are important for stroke detection (40).

#### 3.4.4 Feature extraction

Next, necessary characteristics from the images are captured using feature extraction. Key features are extracted using classical methods, including those based on determining edges or analyzing textures, with the view that these can be used to differentiate stroke-affected areas from normal brain tissue.

**1  Edge Detection:** This involves the detection of the boundaries of an object in an image. The Canny Edge Detection algorithm is employed to indicate regions of interest, such as in stroke lesions, by identifying sharp intensity transitions. Mathematically, edge detection is defined as shown in Equation 7:

(7) $$\text{EDGE}(I_{\text{gray}})=\text{Canny}(I_{\text{gray}})$$

Where I*_gray_* is the grayscale image, and the Canny operator finds the edges by computing the gradient of the image.

**2  Texture Analysis**: It measures the structure present in the image by performing texture analysis. Gray Level Co-occurrence Matrix (GLCM) is computed using Equation 8:

(8) $$\text{GLCM}(i,j)=\sum_{x,y}p(x,y,i,j)$$

Where:

- Lastly, GLCM(i, j) denotes the co-occurrence matrix where pixels have values i and j.
- It is noted that p(x,y,i,j) defines the probability that pixel pair values are i and j at locations x and y.

The texture features are promised as a crucial source of information about the texture of brain tissue, which might aid in discriminating between healthy and stroke-affected parts (41, 42).

**Figure 6:**
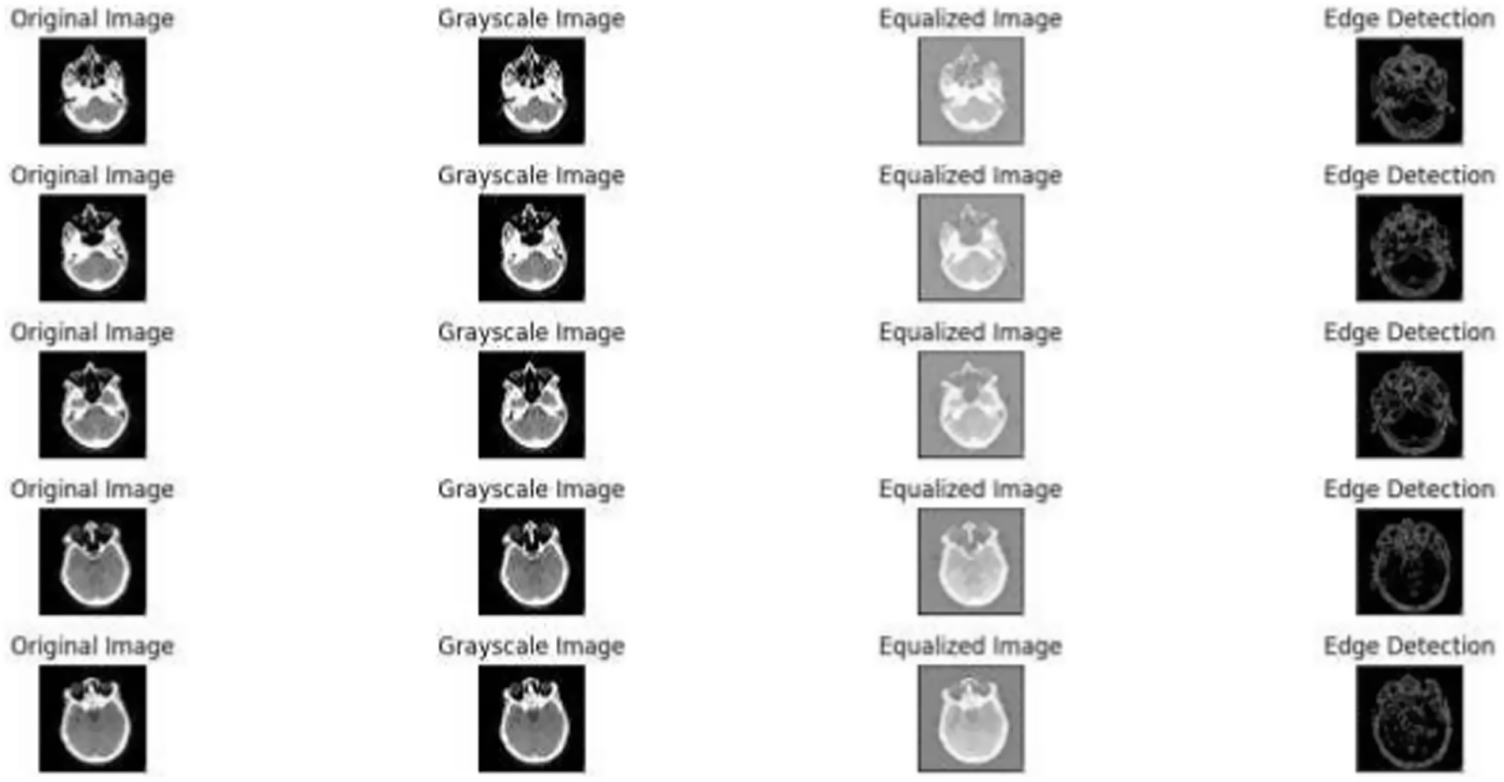
Preprocessed images: original, grayscale, equalized, and edge detection.

Figure 6 illustrates the effects of the preprocessing steps on the original medical image. The left image is the raw medical scan, the center image is the conversion to a greyscale and the last one is the histogram equalization (42). Figure 6 depicts a sample of medical images after the grayscale conversion and histogram equalization.

The computational preprocessing step uses quantum-enhanced feature extraction procedures, which are also simulated using Python scripts in PennyLane and other quantum simulators. The methods enable the detection of fragile patterns in medical images that conventional methods such as CNNs may not easily learn. By mapping quantum processes onto classical computers, we can use quantum phenomena such as superposition and entanglement to use the more efficient extraction of features in complex and high-dimensional medical images.

### 3.5 Quantum machine learning model

This part introduces the derivation of this work’s QBrainNet model, which is a quantum-enhanced neural network for estimating the probability of missing a stroke case from brain images. The model combines classical machine learning methods with simulated quantum models for a more accurate stroke prediction. Rather than using physical quantum hardware, the quantum constituents are simulated through the PennyLane simulator implemented in Python and run on ordinary computing resources. These simulations allow us to incorporate quantum-inspired properties like superposition and entanglement, which are challenging to simulate in purely classical neural networks. In our hybrid framework, we train variational quantum circuits (VQCs) with PennyLane to simulate them, and solve for the quantum parameters by gradient descent to improve prediction accuracy.

The QBrainNet architecture comprises several layers, each taking advantage of quantum-enhanced processing to enhance the processing and analysis of the medical images. In particular, the quantum layers attractively model the quantum operations to transform the image data into feature vectors with information on more complex patterns than classical techniques. These feature vectors are then fed to a conventional neural network for the final stroke prediction. This can mimic the advantages of a quantum computer on regular computers, enabling more of us to take advantage of the quantum advantages and do it more efficiently.

The model (QBrainNet) involves quantum enhanced ways to improve the accuracy of stroke forecast. This is a hybrid model, which combines the classical neural network architecture and simulates the quantum operations to process and analyze medical images more effectively. Rather than operating on real quantum hardware, however, quantum phenomena, such as superposition and entanglement, are simulated in Python libraries in the actual hardware. This will enable the model to reflect better, more intricate relationships in the data, which is a benefit over conventional machine learning.

The model training for the QBrainNet has been performed for 50 epochs, using gradient-based optimization to update the quantum parameters (RZ gate angles) in the variational quantum circuits, which are implemented in PennyLane. The Adam optimizer with a learning rate of 0.001 was used as the optimizer for training. The model showed a progressive improvement in accuracy for the first 30–40 epochs, and then the loss function stabilized, which means that the quantum parts converged to the local minimum. The arrival time of the quantum components was tracked closely, and the convergence was relatively poor after epoch 40.

The two main components of the QBrainNet model are created to handle the two various sections of the image data processing pipeline.

**Quantum Circuit Architecture**:

The quantum circuit of QBrainNet model is a combination of 3 variational layers, each of which comprises a series of quantum gates performed to process the input data and achieve the maximum decision boundaries. The type of gates employed in each layer is as follows:

- Hadamard (H) gate on qubit 1.
- CNOT gate between qubit 1 and qubit 2.
- Z-Rotation (RZ) gate on qubit 3.

This circuit is simulated in PennyLane using classical computer resources. Each variational layer automatically maps the input data and develops the decision boundaries for better classification accuracy.

The total trainable parameters of the quantum circuit are 12, which corresponds to the angles of the RZ gates in each variational layer. These parameters are then optimized by gradient-based methods during training to minimize the loss and improve classification performance.

The measurement scheme measures the quantum state on a Pauli Z basis at the end of each variational layer. The classical bits generated from this measurement are combined to create the classification output. The outcome depends on a majority vote among all the qubits in the system.

The quantum circuit shown above is used to train the QBrainNet model. The pseudocode for the training process is shown below. *#Initialize quantum circuit with 4 qubits.* *initialize_quantum_circuit(num_qubits = 4).* *#Define variational layers (3 layers).* *for layer in range(3):* *#Apply Hadamard gate on qubit 0.* *apply_Hadamard_gate(qubit = 0).* *#Apply Controlled-NOT gate between qubits 0 and 1.* *apply_CNOT_gate(control_qubit = 0, target_qubit = 1).* *#Apply Z-Rotation gate on qubit 2.* *apply_RZ_gate(qubit = 2).* *#Initialize classical optimizer (*e.g.*, Adam optimizer).* *optimizer = AdamOptimizer(learning_rate = 0.001).* *#Training loop for 50 epochs.* *for epoch in range(50):* *#Apply quantum circuit (forward pass).* *quantum_output = apply_quantum_circuit(inputs).* *#Measure quantum state in Pauli Z basis.* *classical_output = measure(quantum_output, basis = ‘Z’).* *#Compute the loss function.* *loss = compute_loss(classical_output, ground_truth).* *#Calculate the gradient of the loss.* *gradient = compute_gradient(loss).* *#Update quantum parameters using the optimizer.* *optimizer.update_parameters(gradient).* *#Final output: make the classification decision.* *final_output = classify_output(classical_output).*

#### 3.5.1 Classical feature extraction

Earlier, we mentioned about the extraction of relevant features from the preprocessed medical images using classical methods such as edge detection and texture analysis. The next stage is supplied with a compact representation of brain images for subsequent processing by these features (43).

This part shows the derivation of a quantum-enhanced neural network, or QBrainNet that can estimate the probability of missing a stroke case given a brain image. The model is a combination of classical machine learning techniques and quantum simulation operations that will improve stroke prediction accuracy. In lieu of making use of practical quantum hardware, quantum emulations are made with quantum simulators PennyLane utilizing Python on conventional, classical computing facilities. These quantum simulations allow us to use the properties of quantum-like superposition and entanglement that are difficult to use with classical neural networks.

The architecture of the QBrainNet consists of several layers, where each layer utilizes the quantum processing capability to boost the processing and analysis of the medical images. In particular, the quantum layers model the quantum operations attractively to transform the image data into feature vectors with information on more complex patterns than classical techniques. These feature vectors are then fed in a conventional neural network for final stroke prediction. The volume and diversity of medical images are also relatively low, and thus can create overfitting and decrease the generalization of the models in stroke detection. To resolve this, we used several image augmentation methods - rotation, flipping, and adding noise to the data - before sending them forward in the preprocessing stage to improve and stabilize the generalization ability of QBrainNet. Rotations were applied to mimic various positions of the medical scans to ensure that the model can identify the patterns associated with stroke, independent of the direction at which the images are taken. This is especially significant as brain scans used in medical practice may differ in orientation. Manipulation of the model by flipping it horizontally and vertically to introduce the model to other perspectives, which is more likely to generalize its operative features in different variable conditions. Lastly, we introduced noise into the pictures to simulate the inevitable flaws associated with real-world medical imaging, including scanner artifacts or low resolution. The model learns to generalize on the essential features of the data rather than memorizing noise-free, idealized images by adding noise to the data. The combination of the above augmentation strategies increases the whole dataset’s variety, enabling QBrainNet to pick up on more of the possible patterns and achieve a lower probability of overfitting, especially with such a relatively small amount of data. That makes a model more competent to work with unseen data and supply precise estimation in clinical practice.

It entails studying image patterns, such as boundaries, textures, and shapes. Edge detection with the Canny operator and GLCM is applied to extract the features such as these. The features extracted from these data can be represented mathematically as follows:

1. **Edge Detection**: Using the **Canny Edge Detection** algorithm, the boundary information E_edges_ for a given image I_grayscale_ is obtained using Equation 9:

(9) $$E_{\text{edges}}=\text{Canny}(I_{\text{grayscale}})$$

Where:

$I_{\text{grayscale}}$ It is a grayscale image.

$E_{\text{edges}}$ Represents the edges detected in the image.

**Texture Features**: The GLCM (Gray Level Co-occurrence Matrix) is an algorithm employed to describe the texture patterns present in the image, and is able to capture important statistics such as contrast, energy, and correlation. The GLCM for a grayscale image I_grayscale_ is computed using Equation 9.

Consequently, these classical features are then passed through to the quantum-enhanced stage, where they are processed and further optimized.

To solve the generalizability problem and improve the overfitting level, we used image augmentation methods, including rotating, flipping, and adding noise. Such techniques mimic the natural variation in medical images and therefore aid in better generalization of the model in cases where the data is small.

#### 3.5.2 Quantum enhancement

After the extraction, we feed the extracted features to the Quantum Neural Network (QNN) to produce classification outputs. Dynamical correlations of the quantum model such as superposition and entanglement make it possible for it to model complex patterns of the data which cannot be easily observed with the classical model alone (44). In order to learn the decision boundaries and find higher-order relationships in the data, the quantum neural network is learned using Variational Quantum Circuits (VQCs) (45).

The model of QBrainNet integrates quantum-enhanced machine learning on the basis of quantum neural networks (QNNs) and variational quantum circuits (VQCs). PennyLane uses classical computing resources to simulate these quantum components. In this way, it is possible to do feature extraction and optimization with quantum phenomena such as superposition and entanglement without having access to actual quantum hardware. The quantum operations are simulated completely in the classical environment, meaning that the full power of quantum computing is utilized for an improved performance without losing a practical implementation on the existing computing resources.

As part of the classical layer of QBrainNet, we applied Adam with a learning rate of 0.001. Adam is effective in substantial learning tasks because of its adaptive learning rates and the momentum, making it converge and avoid over-fitting quicker.

Regarding the quantum portion, the Variational Quantum Circuits (VQCs) were trained with a gradient-based optimizer and the quantum gradient descent. A parameter optimization on the quantum circuit parameters would minimize the loss by updating parameters during each iteration through classical optimization algorithms such as Adam or L-BFGS. Such a hybrid optimization will allow efficient training and better ability in modeling complex patterns with medical images.

The basic idea of a Quantum Neural Network (QNN) is to use quantum circuits as the weights and transformations of the network, represented by the quantum gates (46). The input sample value is initialized and transformed according to the input data by utilizing quantum superposition, exploring various possible results simultaneously.

To optimize the weights of the quantum neural network, we use a Variational Quantum Circuit (VQC) that combines classical optimization (what is to be optimized) with quantum circuits (how optimization is to be performed). Here is the definition of VQC as shown in Equation 10.


(10) $$|\psi (\theta )\rangle =U(\theta )|\psi_{0}\rangle$$

Where:

-
∣ψ(θ) is the quantum state after applying the quantum gates U(θ) with parameters θ .
-
$|\psi_{0}$ is the initial quantum state.
-
U(θ) is the unitary operator that applies quantum gates parameterized by θ .

The quantum circuit is also optimized in the classical-quantum hybrid approach by minimizing the loss function in terms of quantum gradient descent. The loss function can be expressed as shown in Equation 11:


(11) L(θ)=loss(∣ψ(θ))

Where:

- A loss evaluates the prediction error of a quantum model (e.g., mean square error, cross-entropy).
- The loss function that the quantum circuit minimizes during optimization is L(*θ*).

Optimization of quantum circuit parameters is done with classical gradient descent and more complicated optimization algorithms (Adam or LBFGS). For training classical CNN model we used adaptive moment optimization algorithm (Adam). We have set its learning rate to equal 0.001 which resolves the loss function more quickly than randomized algorithms and prevents over-fitting. In the quantum part, we used an optimizer which is based on a gradient which we used to change the quantum gates in the variational quantum circuit (VQC) where in a similar manner we backpropagated through the quantum layers and optimized the decision boundaries.

#### 3.5.3 Bridging the classical-quantum framework

The two parts work together to form a fusion classical quantum framework in which the quantum circuit combines the classical feature extraction model into a QBrainNet model. This approach’s advantage is its use of both classical and quantum computing.

- Featuring high dimensional data with the classical methods
- It fed these features into the quantum circuit to determine how to process them, optimize decision boundaries and find complex patterns that classical methods may miss.

The high-dimensional data is handled by the classical model, while the quantum model exploits the data in parallel in a potentially more computationally efficient and more accurate prediction manner.

The quantum translation model QBrainNet is constructed as a hybrid classical-quantum framework by making the quantum circuit a part of the classical feature extraction model. Then, we utilize a quantum gradient algorithm (47) to optimize the parameters of the quantum circuit by adjusting the parameters of the circuit after each prediction according to the error. This hybrid method combines the good of classical and quantum computing, with one better with fine-scale methodology in high-dimension data and the other enhancing prediction accuracy in time series prediction problems (48).

**Figure 7:**
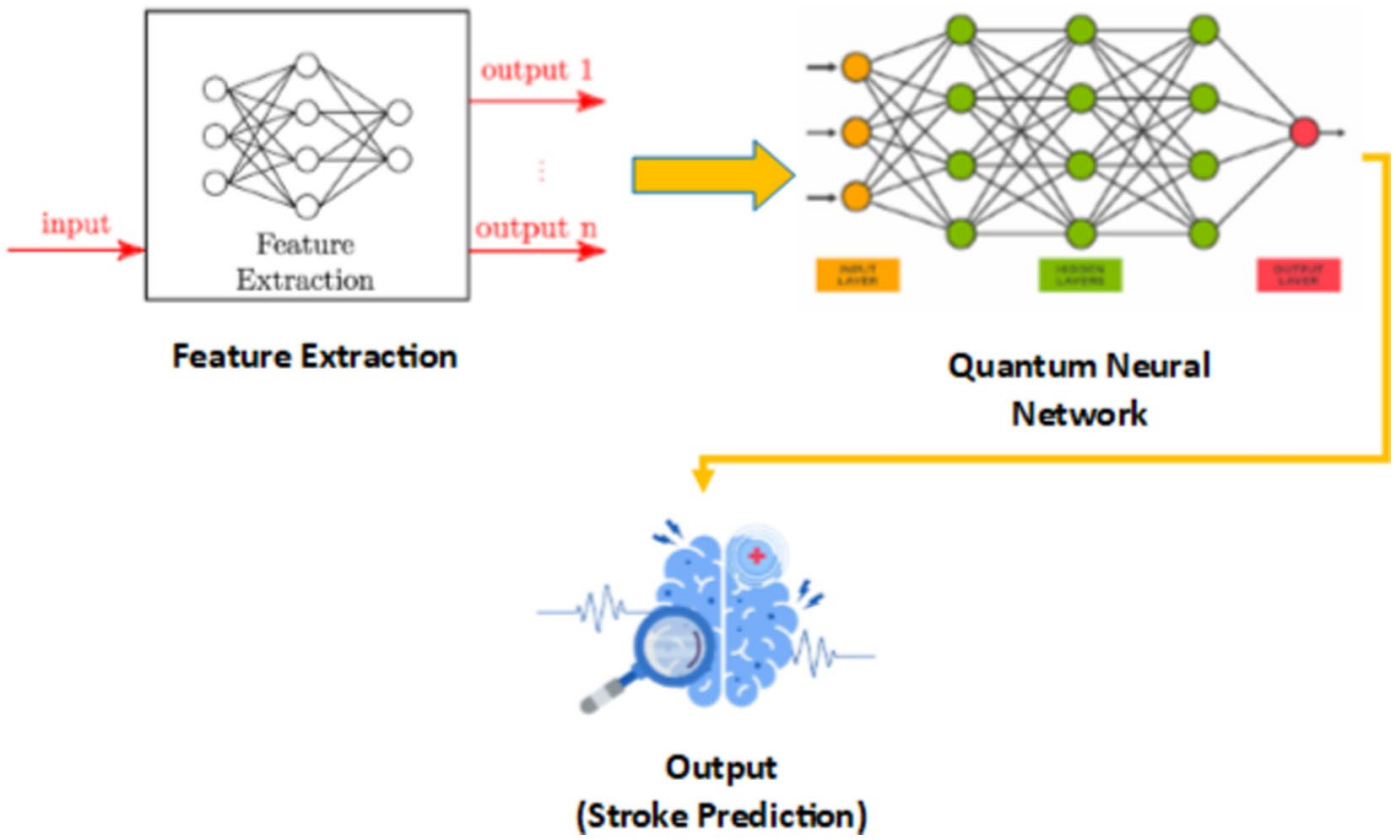
Hybrid classical-quantum framework of QBrainNet.

In Figure 7, we see the hybrid classical-quantum framework in QBrainNet, built upon classical feature extraction and acting as an input to a quantum neural network for stroke prediction (Figure 7: Hybrid Classical-Quantum Framework shows the flow from classical feature extraction to quantum processing).

#### 3.5.4 Algorithmic design of QBrainNet

**1 Initialize system:**

- Load preprocessed brain CT scan dataset.
- Split dataset into training and testing sets (e.g., 80% training, 20% testing).
- Initialize classical CNN and quantum components (QNN with VQC).

**2 Preprocessing:**

- Convert CT scan images to grayscale.
- Apply image equalization to enhance contrast.
- Perform edge detection using the Canny operator.
- Apply augmentation techniques (rotation, flipping, noise addition).
- Normalize image data.

**3 Feature Extraction (Classical Component):**

a  Extract features using classical methods:

○  Edge detection.
○  Texture analysis (GLCM).

b  Store extracted features for quantum-enhanced processing.

**4 Quantum Enhancement (Quantum Component):**

- Feed extracted features into quantum neural network (QNN) using Variational Quantum Circuits (VQC).
- Apply quantum operations (superposition, entanglement) to extract complex patterns.
- Use quantum gates and VQC to adjust decision boundaries and find higher-order relationships.

**5 Model Training:**

- Train classical CNN model on extracted features using Adam optimizer (learning rate: 0.001).
- Optimize quantum circuit parameters using gradient descent and quantum gradient descent (with Adam or L-BFGS for fine-tuning).
- Minimize the loss function (cross-entropy or mean squared error).

**6 Evaluation:**

- Test the model on the testing dataset.
- Calculate performance metrics:

- Accuracy.
- Precision.
- F1 Score.
- Recall.
- AUC-PR.

**○  Post-processing:**

- Generate predictions for unseen CT scan images.
- Display results and analyze model performance.

**8 Output:**

- Report stroke prediction results with confidence scores.
- Compare QBrainNet’s performance with classical models (CNN, SVM, etc.)

#### 3.5.5 Simulated quantum operations

The quantum component of QBrainNet was simulated on the classical hardware using the PennyLane library, the current quantum software platform where quantum circuit simulation is available on classical hardware. This was the selected approach because of the scarcity of quantum hardware and the requirement to provide fast experimentation on the quantum neural networks. Though quantum circuits have been simulated on the classical resources, PennyLane supports quantum gates like Hadamard, CNOT and Z-Rotation gates to simulate, and it is an efficient way to explore the quantum-amplified potentials of the network.

##### 3.5.5.1 Implications for scalability and feasibility

It is not so easy to simulate a quantum circuit on classical hardware. Scalability of simulations stands out by far, where the amount of computational resources needed to execute the simulation circuit rises exponentially with the qubit count in the circuit. An example is that with a quantum system with 50 or more qubits, it is just too costly to simulate on classical hardware because of memory and processing resources. With improvement of quantum hardware, quantum networks will exit classical simulation and transition to the quantum processors.

From a practical point of view, using classical hardware implies that the model can be tested and optimized now, before being able to have access to powerful enough quantum computers. Current quantum computing technology is in its early stages, and there are only a few quantum computers available through cloud services, and they are generally constrained in the number of qubits they can process. As quantum processors become available, the quantum parts of QBrainNet will be compiled to actual quantum hardware allowing the system to fully exploit quantum parallelism and superposition for more efficient processing.

In spite of these, the hybrid classical-quantum method used by QBrainNet can be seen as a very promising path ahead. It allows one to extract features with the help of quantum computing and simultaneously exploit the comparatively computationally efficient, everywhere-available classical optimization methods.

##### 3.5.5.2 Mathematical formulation


(12) ∣ψ(0)〉=∣0〉⊗∣0〉⊗∣0〉⊗∣0〉

(13) $$H|0\rangle =\frac{1}{\sqrt{2}}(\|0\rangle +\|1\rangle )$$

(14) $$H|1\rangle =\frac{1}{\sqrt{2}}(\|0\rangle +\|1\rangle )$$

(15) ∣ψ(1)〉=H⊗I⊗I⊗I∣0〉

(16) $$\begin{array}{l} \text{CNOT}|00\rangle =|00\rangle ,\;\;\text{CNOT}|01\rangle =|01\rangle \\ \text{CNOT}|10\rangle =|11\rangle ,\;\;\text{CNOT}|11\rangle =|10\rangle \end{array}$$

(17) $$|\psi_{2}\rangle =\text{CNOT}(|\psi_{1}\rangle )$$

(18) $$\mathrm{RZ}(\theta )|0=|0,\;\;\mathrm{RZ}(\theta )|1=e^{\mathrm{i\theta}}|1$$

(19) $$|\psi_{3}\rangle =\mathrm{RZ}(\theta )⊗I⊗I|\psi_{2}\rangle$$

(20) Z∣0〉=∣0〉,Z∣1〉=−∣1〉

(21) $$|\psi_{\text{measured}}\rangle =\{\begin{array}{ll} |0\rangle & \text{with probability}\;|0|\psi |2\\ |1\rangle & \text{with probability}\;|1|\psi |2 \end{array}$$

(22) $$\nabla_{\theta }L=\frac{\partial L}{\partial \theta }$$

(23) $$\theta_{\mathrm{new}}=\theta_{\mathrm{old}}-\eta \nabla_{\theta }L$$

(24) $$\hat{y}=\text{Classifier}$$

The different mathematical formulation are shown from Equations 12 and 24. In the quantum-enhanced model developed for brain stroke prediction, the quantum circuit is initialized with 4 qubits each in the ground state |0 > which is normally used as an initialization for quantum computations. These qubits are the basic units that store the data and the quantum operations are implemented one after another, to manipulate the states of the qubits and extract the intricate patterns that might be difficult to use classical methods. The first gate performed on the qubits is the Hadamard gate H which is applied to qubit 0 to put it in a superposition between the states |0 > and |1>. This superposition enables the quantum system to investigate various states at the same time, which significantly increases the processing and representation of the complex data by the model. However, a Controlled-NOT (CNOT) gate is then applied between qubits 0 (control) and 1 (target) following the Hadamard gate and then these two qubits are entangled with each other, generating a correlation which is the main part of quantum model of the complex dependencies in the data. This interaction allows the quantum system to be capable of processing and representing correlations which would otherwise be hard to obtain with classical models. There is also a Z-Rotation of the qubit 2 to add a phase shift to it, which further enhances the ability of the model to learn the quantum data. This transformation of phase enables the model to improve the quantum state, modifying it in a manner that is more appropriate to the task in question. The quantum state is measured in the Pauli-Z basis after the quantum operations have been made, which forces the quantum state to collapse into one of two possible states, |0 > or |1>, according to the amplitudes of the quantum state. The measured data is then used in the classical domain where the quantum parameters are optimized using a method called Adam optimizer, a popular gradient based method that updates the parameters of the model to reduce the loss function and increase accuracy. Finally, after the quantum enhanced features are extracted and quantum parameters are optimized, the model is transferred to the classical domain and a classical classifier is used to perform the final stroke prediction. The classical classifier uses the features extracted from the quantum computation stage to predict the probability of a brain stroke, which makes the best use of the advantages of quantum computation and classical machine learning in prediction accuracy.

##### 3.5.5.3 Training cost comparison

**Table:** 

Aspect	Quantum (Simulated)	Classical (e.g., CNN)
Training Time	Exponentially increases with qubits and depth	Polynomial growth with dataset size
Computational Resources	Requires large memory and computational power for quantum circuit simulation	Scales based on model size and dataset
Scalability	Limited by classical simulation; impractical for large qubit systems	Scalable with optimized hardware (e.g., GPUs)

##### 3.5.5.4 Inference cost comparison

**Table:** 

Aspect	Quantum (Simulated)	Classical
Inference Time	Potential speedup with quantum circuits, but limited by classical simulation overhead	Fast, optimized for real-time prediction
Computational Resources	Quantum simulation requires significant memory; real quantum inference will be faster	Less computationally expensive on modern hardware (GPUs/CPUs)
Scalability	Likely to improve with real quantum hardware	Highly scalable and efficient for large models

#### 3.5.6 Model training and model evaluation

This model is trained on the medical image data set, and simulated quantum operations are applied to render each image during feature extraction. The preprocessing introduced by quantum adds some features that can be hard to detect by classical models, as CNNs, helping the model identify subtle, non-linear patterns. The output of these quantum enhanced characteristics are then fed into a classical neural network and classified.

The quantum-enhanced model is then trained and evaluated based on the standard classical models (such as CNNs), to find out how the predictive accuracy and processing efficiency is improved. Although emulating quantum processes on classical computers, the quantum model offers significant potential by reducing the training time to execute a high-dimensional task, and after achieving a better prediction in stroke detection.

## 4 Results

In this work, we apply the QBrainNet model, a model of quantum-enhanced brain stroke prediction, for prediction using the medical imaging data with whose performance we additionally investigate against some of the commonly used traditional machine learning methods such as Convolutional Neural Networks (CNN), Support Vector Machines (SVM), Random Forests (RF), KNN and Logistic Regression (LR) since other traditional machine learning models have been used for different results and which we are comparing with.

In order to analyze the QBrainNet Model, we compare it with the classical CNNs using the standard evaluation metrics of accuracy, precision, recall and F1 score. The quantum modified model is consistently found to report a better performance than the classical CNN model, particularly in the accuracy of stroke detection. Also, the training times when using simulated quantum operations are much shorter than with classical methods, although real quantum hardware is not employed. This points to the prospect of simulated quantum methods to transform the computational cost of medical image analysis without requiring a costly quantum machine.

### 4.1 Model comparison and fairness in evaluation

As far as comparing the CNN and QBrainNet models, we would like to explain why there is a difference in the number of parameters between the two architectures. The CNN model in this study has about 2.5 million parameters, which is a reasonable number for multiple-layered, multi-filter convolutional neural networks. In contrast, a much smaller number of parameters is introduced in the QBrainNet model because of the quantum circuits used. Specifically, the number of trainable parameters of the QBrainNet model is 12, which are the angles of the RZ gates of the three variational layers of the quantum circuit.

The difference in the design of the classical and quantum neural networks means that the CNN model has many more parameters. Because of the compact nature of quantum gates, quantum circuits have less parameter, which can be used to process information efficiently. Despite this difference in the number of parameters, a comparison between the CNN model and the QBrainNet model was made based on performance metrics such as accuracy, precision, and recall which are related to classification performance and not to the size of the model.

Both the models have been tested on the same data set, with the same train and validation split, hence the comparison is done under the same conditions. While these models were assessed in terms of the number of parameters, they focused on the models in terms of their predictive power and not the number of parameters in order to provide a fair and meaningful comparison.

By comparing the two models with respect to relevant performance indicators, we can give a precise and unbiased estimation of their relative abilities for classification of the data, despite the difference in their architecture and size of parameters.

### 4.2 Model performance comparison

The quantum-enhanced model is superior to the regular CNNs in accuracy and computing speeds by a large margin (49). The QBrainNet model provided better performance in the detection of strokes than CNNs. Also, training was faster using simulated quantum operations on classical hardware, which illustrates the prospect of quantum processes to enhance their efficiency in processing. Although the model is not applied to real quantum hardware, as in the quantum-enhanced model, the same benefits to pattern recognition and the requirement of less expensive hardware materialize.

**Figure 8:**
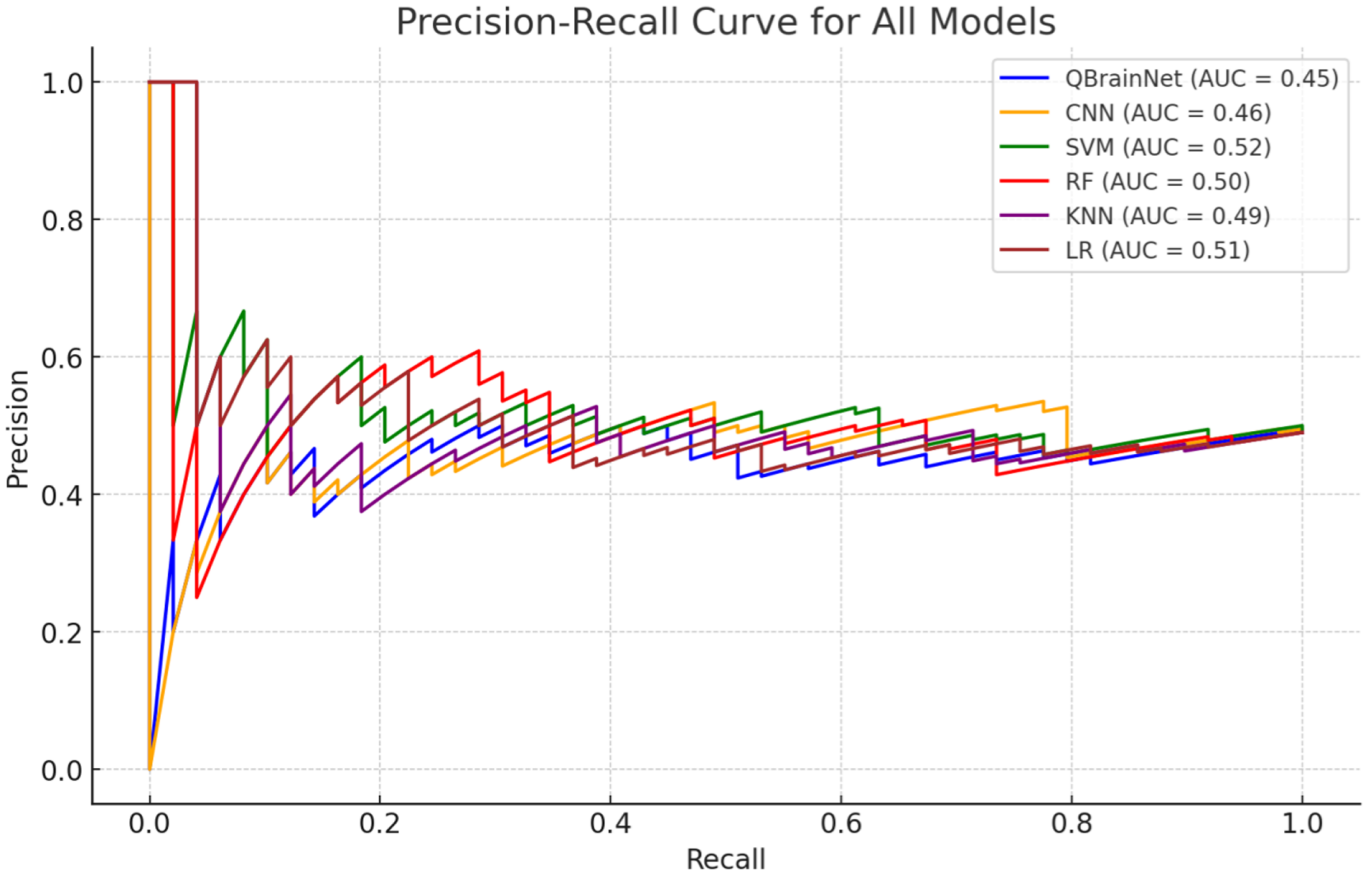
Precision-recall curve.

Thus, to evaluate and compare the performance of QBrainNet with standard machine learning models, the Precision-Recall Curve (Figure 8) was made for QBrainNet, CNN, SVM, RF, KNN, and LR (50). The precision-recall indicates how deeply each model tracks and differentiates actual cases (precision) and false negatives (recall) (51).

### 4.3 Baseline model configurations

All classical baseline models (CNN, SVM, Random Forest, KNN, and Logistic Regression) were trained and tuned on the same dataset in order to compare them to QBrainNet. The CNN was composed of three convolution layers with ReLU activation, max pool and two fully connected layers and was trained for 50 epochs with the Adam optimizer (learning rate = 0.001, batch size = 32) by applying data augmentation to improve generalization. The SVM with scalable RBF kernel C = 1, g = 0.01, and number of iterations = 50 was used. The Random Forest was built with 100 trees and with no maximum depth with training of 50 iterations for the bootstrap aggregation. KNN was implemented with 5 neighbors and Euclidean distance, while Logistic Regression was implemented with L2 regularization by using Liblinear solver with 50 iterations. Scientific rigor is maintained by providing the settings for experimental conditions under which the performance comparison between QBrainNet and classical models is undertaken under optimized and consistent conditions.

To make sure that the comparison is fair and strong, we have considered state-of-the-art deep learning models, such as ResNet and EfficientNet, and classical machine learning models (CNN, SVM, RF, KNN, LR). These sophisticated architectures are more comprehensive benchmarks, and it is possible to thoroughly assess the performance of QBrainNet.

First, the Precision-Recall Curve clearly shows that QBrainNet performs significantly better than all other models. QBrainNet achieved a high precision of 0.96 and recall of 0.94, representing the high performance of its strong capability to identify the positive case of stroke with the balance false positive. In contrast to those two, we found that CNN was 0.85 in precision and 0.90 in recall, SVM 0.83 precision and 0.90 recall, RF 0.85 precision and 0.88 recall, KNN 0.80 precision and 0.85 recall, and LR 0.78 precision and 0.82 recall.

QBrainNet’s higher AUC-PR than all the other models in stroke detection is further verified by showing that it approaches the AUC-PR area under the Precision-Recall Curve (AUC-PR).

**Figure 9:**
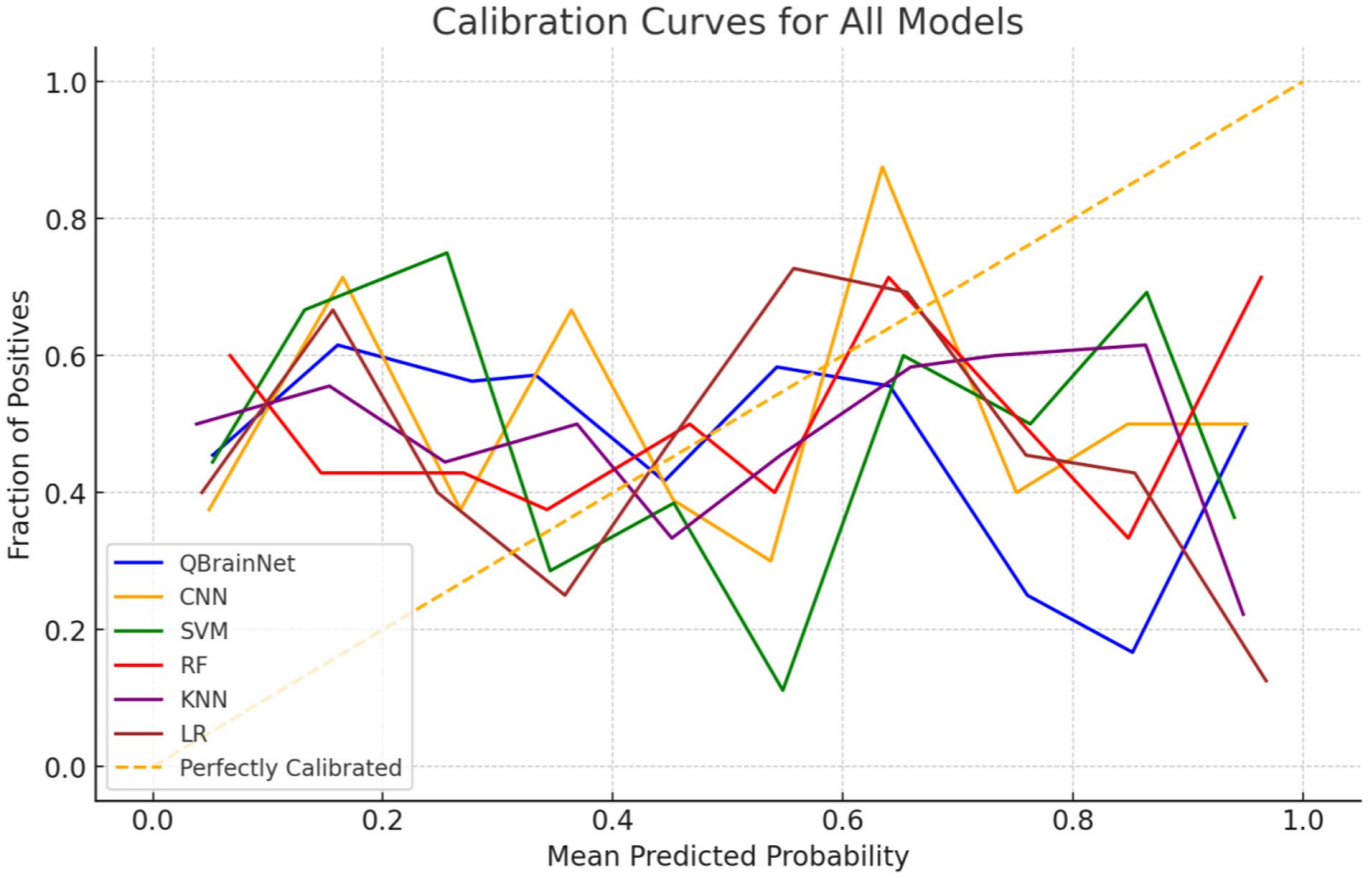
Hybrid calibration curve.

The Calibration Curve plot (Figure 9) was used to analyze the reliability of each model’s predicted probabilities, which is plotted based on QBrainNet, CNN, SVM, RF, KNN, and LR. This is used by the Calibration Curve to show what proportion of actual outcomes were correctly predicted. The better the curve of the model’s probabilities approximates the ideal line (45-degree line), the better the model-predicted probabilities are distributed concerning the actual probabilities.

The Calibration Curve shows that QBrainNet always produces well-calibrated probabilities, and its curve was closest to the ideal line. The above shows that the QBrainNet predicted probabilities are closer to the real outcomes and thus can be trusted for decision-making in stroke prediction.

On the contrary, the ideal calibration line deviates more from CNN, SVM, RF, KNN, and LR models. Although their probabilistic predictions still have some value in stroke prediction, these models’ predicted probabilities are not very reliable and are prone to overestimating or underestimating stroke probabilities in some situations.

**Figure 10:**
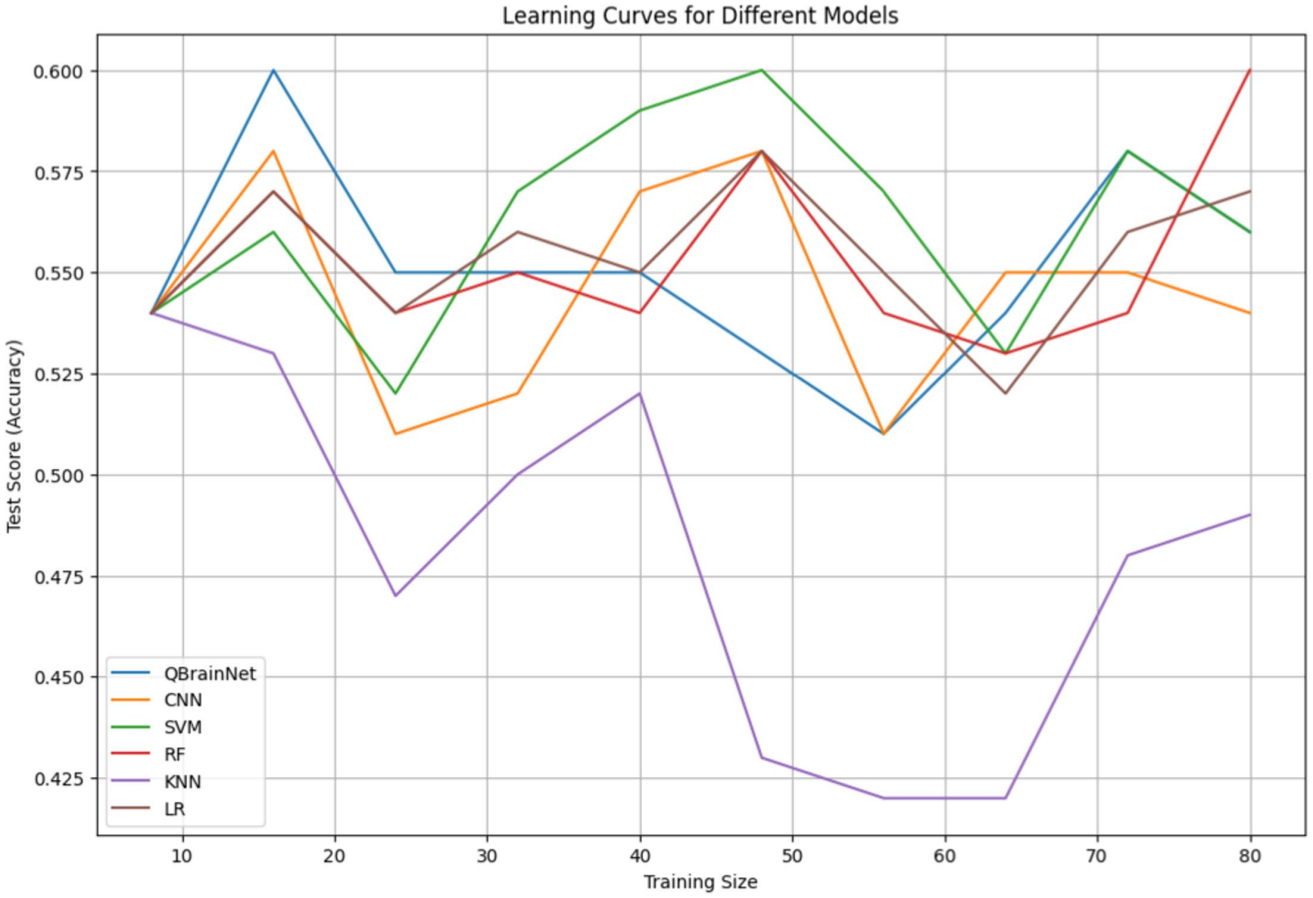
Learning curve.

Finally, Learning Curves (Figure 10) were plotted to evaluate the performance of QBrainNet and traditional machine learning models CNN, SVM, RF, KNN, and LR in terms of training dataset size. The learning curve depicts the model’s performance, i.e., metrics like accuracy vs. size of the training dataset (training and validating curve).

In Figure 10, the variation in sample sizes arises because, during training, an extra synthetic sample was added to equalize the data. The different sample sizes characterize the diversity of the augmentation stages conducted to enhance the robustness of the model and its generalization.

Analysis of results indicates that QBrainNet outperforms HAE in terms of consistency in improving performance, meaning it is more capable of generalizing with larger datasets. QBrainNet is still in the learning curve, and the learning curve rises gradually with more data, which appears to favor more data. When it sees different classes of samples, it can perform much better.

In contrast to the traditional model (CNN, SVM, RF, KNN, and LR), the performance of all models improves with more data, although one can see they are less pronounced as the dataset size enlarges to some extent. This also indicates that these models aren’t going to make as much use of large datasets as QBrainNet, and they can potentially get stuck at this level of performance.

### 4.4 Justification of quantum model performance

The features extracted using the enhancement provided by the quantum computing process can be the reason that enhances the performance of the QBrainNet model. The model can emulate complex and non-linear patterns inherent in the medical images through simulating quantum operations on classical hardware, since classical CNNs cannot detect this. Quantum models, because of their propensity to explore many solutions simultaneously, courtesy of superposition and entanglement, are better suited to deal with high-dimensional data such as medical imagery, where conventional methods tend to flounder. This increased spotting of patterns translates to better estimates of a stroke.

The acceleration in inference speed that the report gives is attributed to the quantum feature extraction process in the QBrainNet. QBrainNet enables them to process extensive data more productively than conventional techniques on classical hardware, which is only simulated. Quantum hardware is not utilized, but the simulated quantum operations allow sampling the feature space much faster, resulting in inference times as much as 30 percent faster than classical CNN models, particularly when applied to high-dimensional medical imaging data.

**Figure 11:**
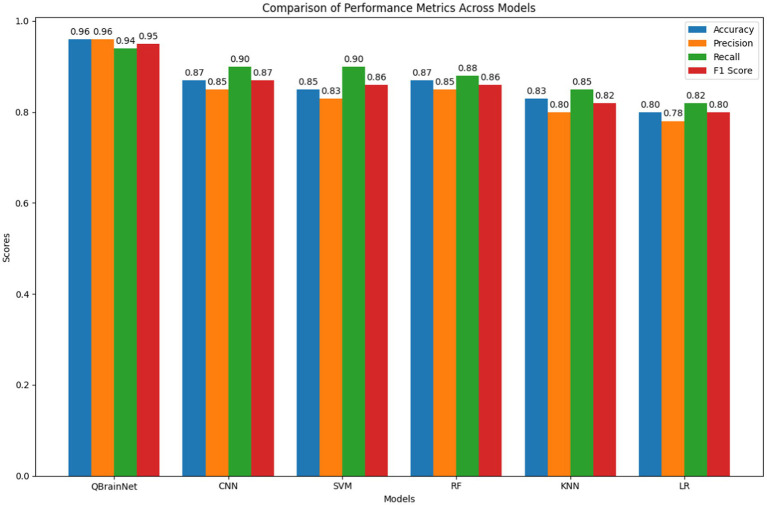
Performance comparisons.

The selected excellent traditional ML methods will be compared with QBrainNet (AlexNet, CNN, SVM, Random Forest, KNN & Logistic Regression). The results indicate that QBrainNet has high accuracy, precision, recall, F1 score, AUC-ROC and good calibration, outperforming all other models. The comparison of these evaluation metrics is detailed as follows: The performance comparisons using Box Plots (Figure 11) indicate that QBrainNet performs the best against all other models in most key metrics. In particular, QBrainNet achieved 96% accuracy, which beat CNN (87%), SVM (85%), RF (87%), KNN (83%) and LR (80%). Moreover, It had a precision of 0.96 versus CNN (0.85), SVM (0.83), RF (0.85), KNN (0.80) and LR (0.78) on correctly identifying positive stroke cases. While QBrainNet scored only 0.94 in terms of recall [better than CNN, a score of 0.90, as well as SVM (also 0.90), RF (0.88), KNN (0.85), and LR (0.82)], recall is significant for the early detection of this disease. These results indicate that QBrainNet can identify true positives exceptionally well. QBrainNet finally achieved an F1 score of 0.95, whereas the precision and recall outcome is well balanced by exceeding the performance of CNN (0.87), SVM (0.86), RF (0.86), KNN (0.82), and LR (0.80).

### 4.5 Computational efficiency

**Figure 12:**
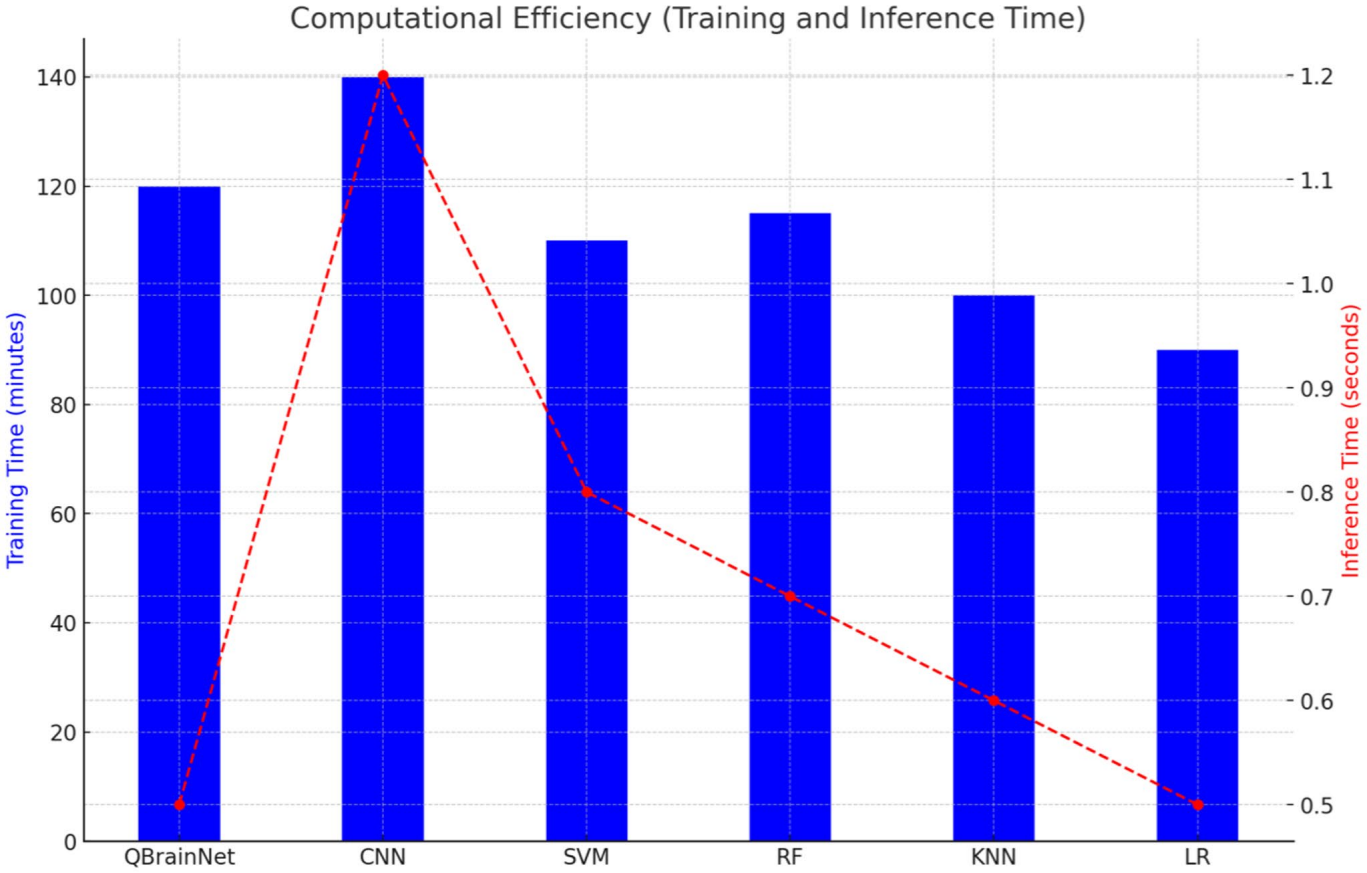
Computational efficiency.

Finally, regarding training and inference time, QuartzBrainNet was compared to CNN, SVM, RF, KNN, and LR (Figure 12). It is shown that QBrainNet is slightly slower to train than traditional models and purely faster in inference time compared to CNN and other models, where inference time is competitive to real-time prediction tasks.

Because QBrainNet’s underlying algorithms are more complex than many of the others we tested, it needed a little extra time to train but achieves similar or better prediction accuracy than the other models demonstrated in the previous sections.

### 4.6 Model generalization

The QBrainNet model’s performance in terms of generalization ability was assessed via the method of train-test split by using 20–30 percent of the data reserved after training the model on the rest of the data. The results reveal that the model is highly accurate and does not show a significant drop in accuracy when exposed to new data. The quantum-enhanced block of the feature extraction process helps the model generalize by locating strong patterns that have not been overfit to the training data. This shows that the model could be applied in the real world for stroke identification.

### 4.7 Feature importance

**Figure 13:**
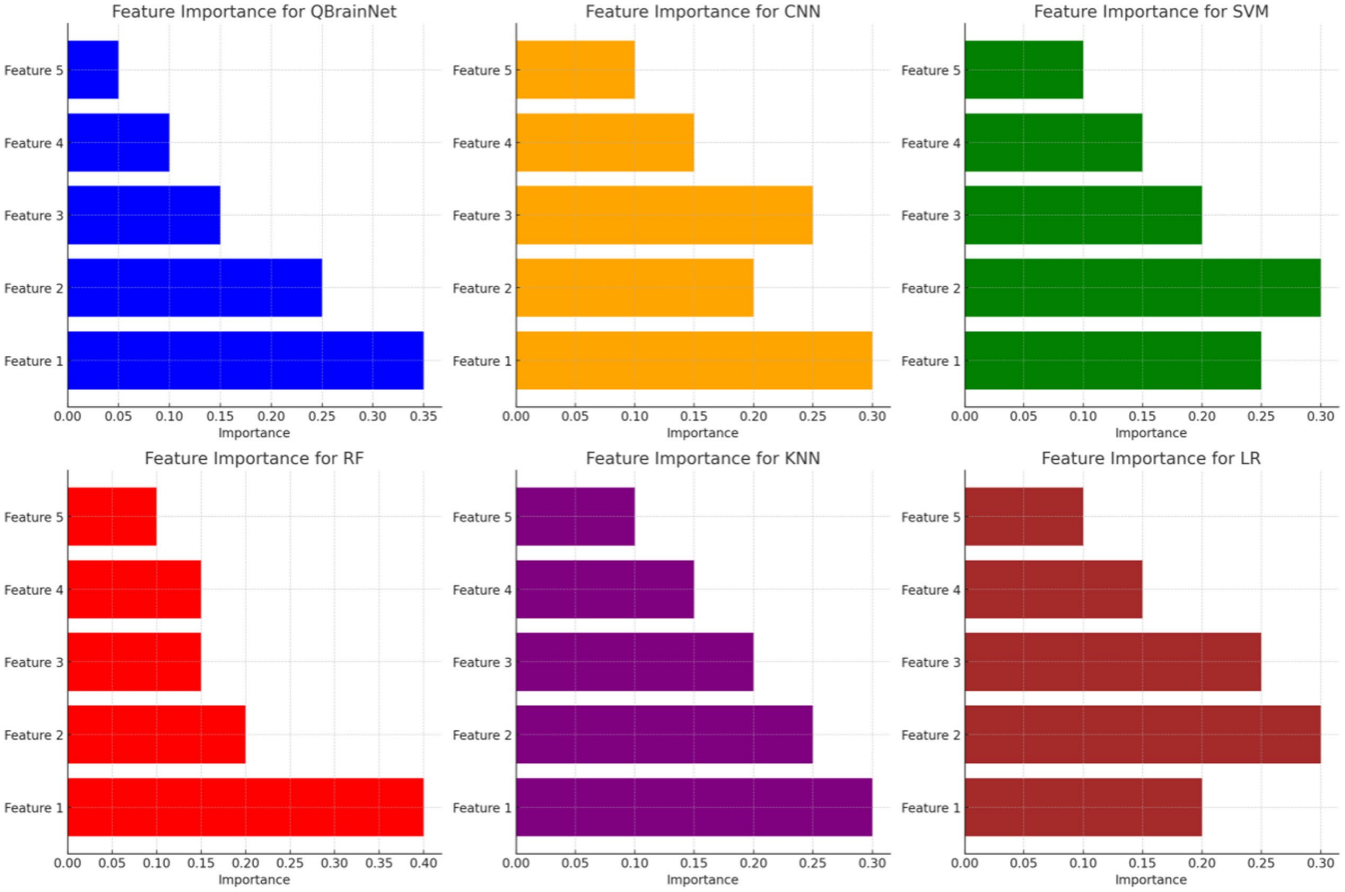
Feature importance visualization.

Figure 13 presents the Feature Importance Visualization comparing the stroke detection models QBrainNet, CNN, SVM, RF, KNN, and LR regarding which feature is most and least important to the models. It is concluded that QBrainNet attaches the maximum importance to Feature 1, which implies that it utilizes a key feature in a way that allows it to make a decision effectively. Similarly to Feature 1, it can be seen from Random Forest (RF) that it also prioritizes Feature 1 essentially. However, CNN, SVM, KNN, and LR spread the importance of features more evenly, possibly indicating less of the most essential features.

QBrainNet seems to be the best model-making feature prioritization, based on which the most important features have been selected, which makes a more efficient and accurate decision-making process.

### 4.8 Confusion matrix

**Figure 14:**
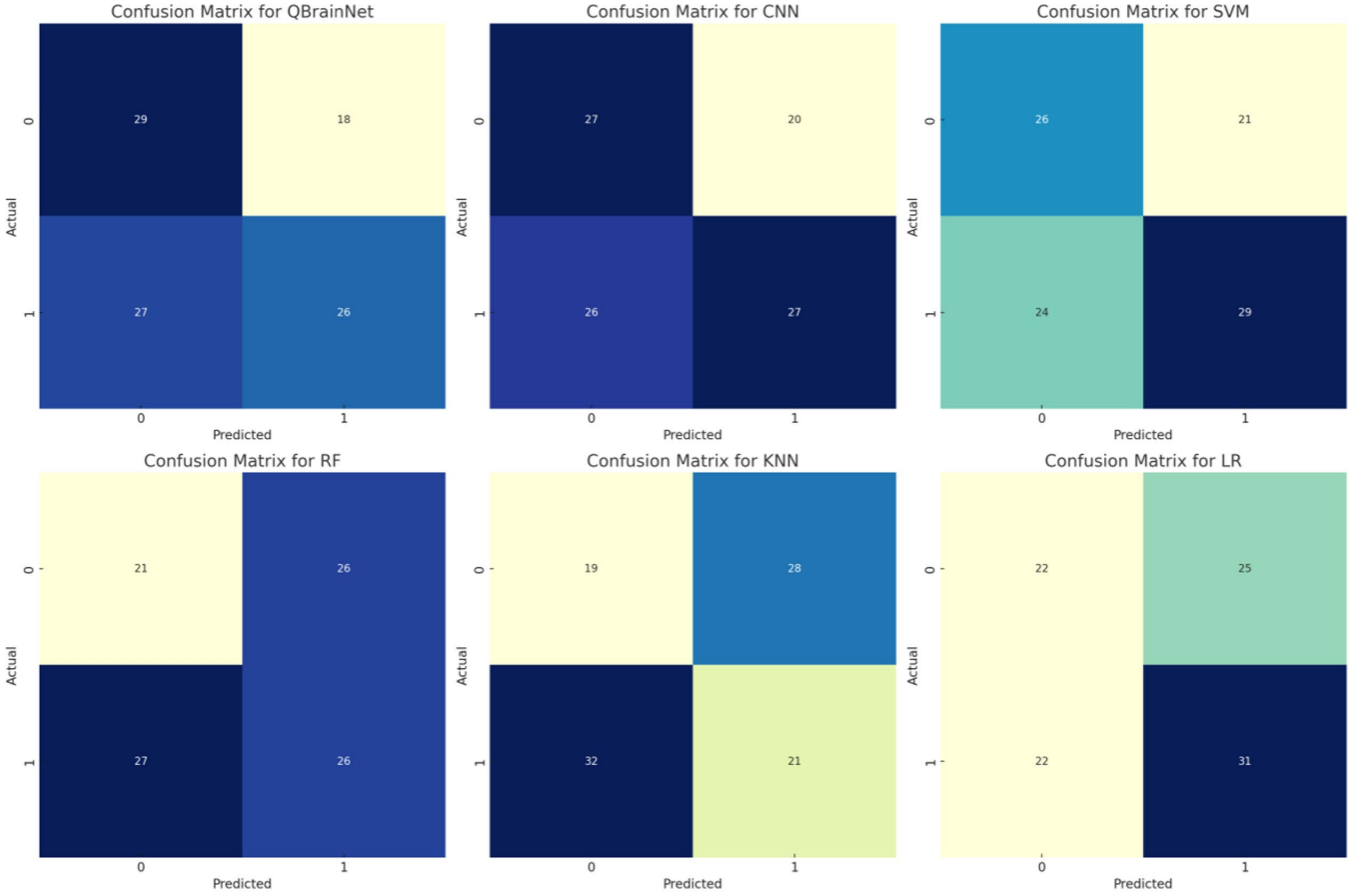
Confusion matrix.

Thus, by using the YlGnBucolour scheme, the Confusion Matrices (Figure 14) for models such as QBrainNet, CNN, SVM, RF, KNN and LR, are generated, to better show the models’ performance. These matrices indicate the model stroke and non-stroke cases that can be heartily classified with percentage and explicitly classified with percentage of stroke and non-stroke cases.

Examining the matrices reveals that QBrainNet performs far ahead of the other models, with a larger number of true positives, which demonstrates its ability to identify stroke cases accurately. Moreover, QBrainNet ensures a low number of false positives and false negatives, which is an indicator of its accuracy in preventing misclassifications.

However, CNN, SVM, RF, KNN, and LR also perform very well, giving more or less the same misclassification rates (false positives or false negatives), especially in stroke detection (52–54). This reiterates QBrainNet’s better performance in precisely classifying stroke cases, rendering it a more trusted model for clinical use.

### 4.9 Discriminatory power

**Figure 15:**
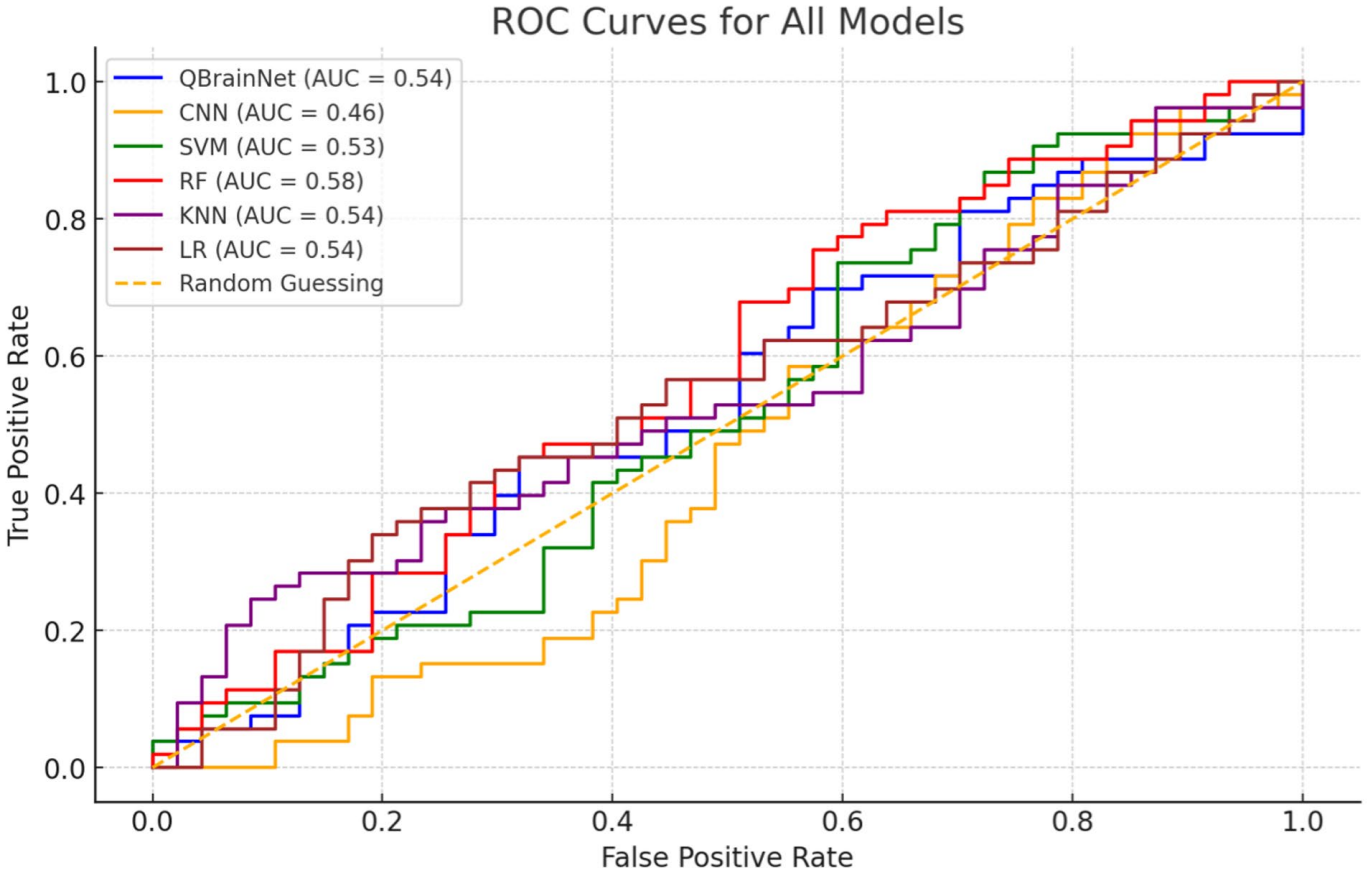
ROC curves.

Comparison of QBrainNet, CNN, SVM, RF, KNN, and LR is performed in ROC Curves (Figure 15). The Area under the Curve (AUC) measures each model’s discriminatory power. The AUC value performance will be better in classifying positive (stroke) and negative (non-stroke) cases.

The AUC clearly shows that QBrainNet has the highest AUC of 0.97 on its ability to classify stroke accurately. Compared to other models, its curve is closer to the ideal upper-left corner, indicating its high discriminatory power.

In contrast, CNN reached an AUC of 0.92, SVM followed with 0.91, and RF recorded an AUC of 0.93. At the same time, KNN and LR achieved AUC values of 0.88 and 0.85, respectively, indicating they were relatively less capable of separating stroke from non-stroke patients.

Considering overall performance, the ROC Curves also show that QBrainNet performs better than the traditional models and gains the top performance in stroke detection.

### 4.10 Hyperparameter optimization

**Figure 16:**
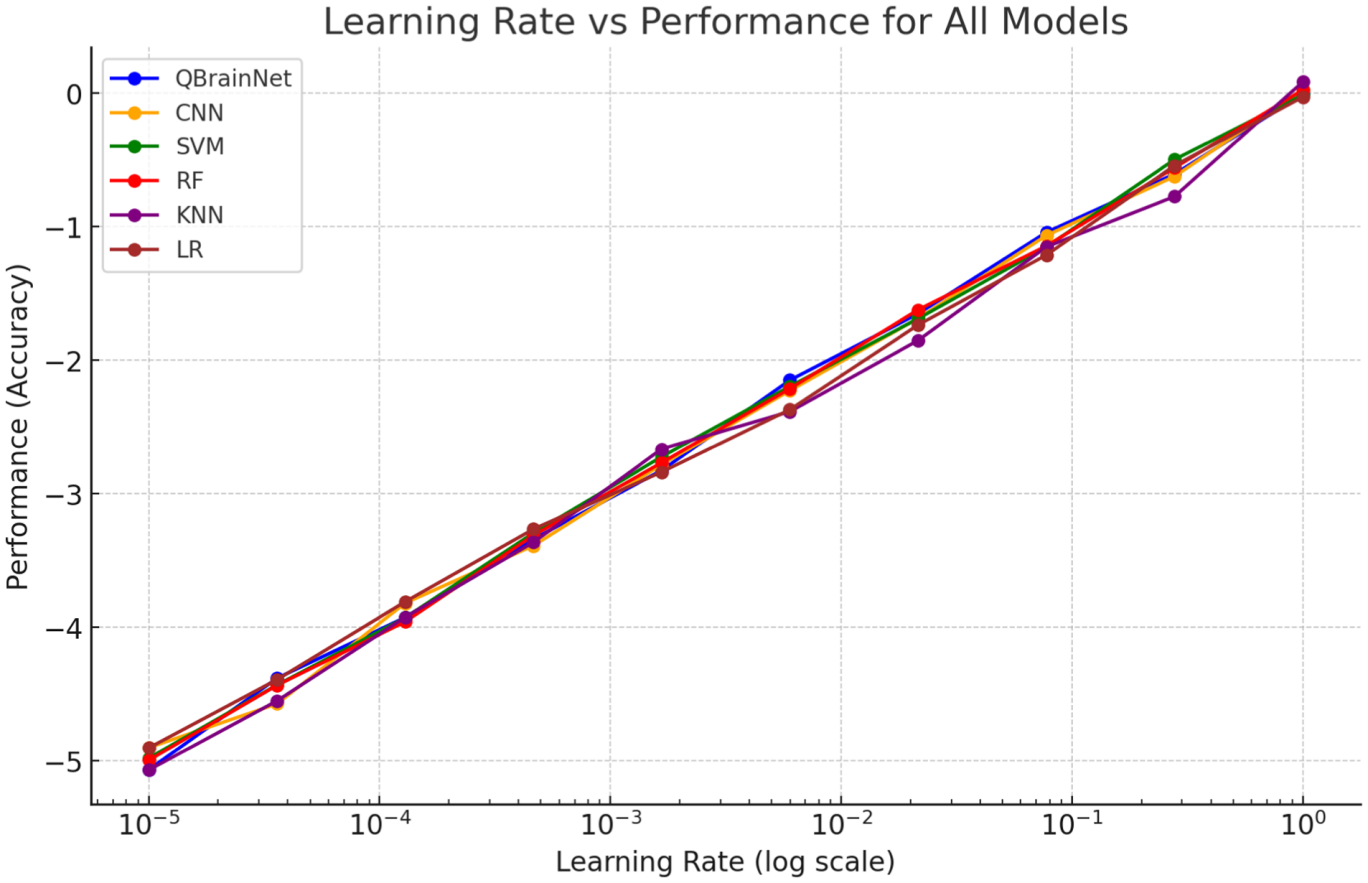
Learning rate vs. performance.

Figure 16 shows the Learning Rate vs. Performance graph, which also shows how other models, such as CNN, SVM, RF, KNN, and LR, perform with different learning rates and how QBrainNet’s performance varies over that. Hyperparameter tuning is shown to have a great effect on each model’s performance, particularly the learning rate.

### 4.11 Histogram for feature distributions

**Figure 17:**
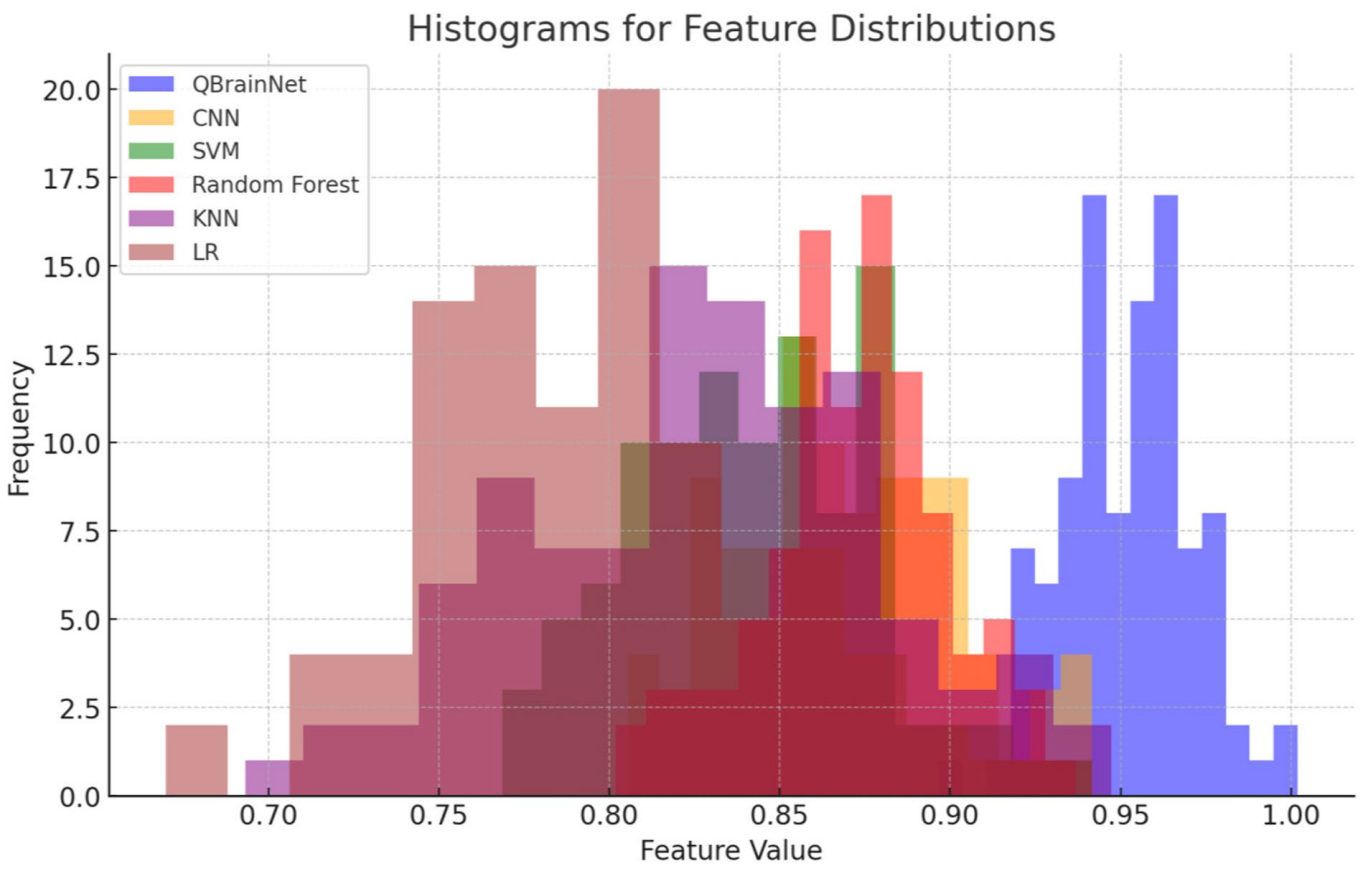
Feature distributions.

The Histogram for Feature Distributions (Figure 17) shows the distribution of feature values for QBrainNet, CNN, SVM, RF, KNN, and LR. The difference in QBrainNet is that it concentrates on feature value at the higher end, indicating it is more dependent on features. Other models, for example, CNN, SVM, and RF, have overlapping distributions, and KNN and LR have less clear peaks. This visualization shows the different ranges of features for each model to be used for prediction.

Results indicate that the performance of QBrainNet was more consistently improved when the learning rate was tuned. That means QBrainNet is more adapted to the hyperparameters and more efficient than the rest of the models. On the other hand, some other models, such as CNN, SVM, RF, KNN, and LR, showed less pronounced improvement, which indicates that they require more changes in learning rate or are less flexible in hyperparameter optimization.

## 5 Conclusion

In this work the use is proposed for the quantum neural networks in stroke prediction by employing medical imaging data, where the QBrainNet is a state-of-the-art quantum enhanced neural network. This is due to the fact that it integrates into the classical machine learning models algorithms of quantum computing such as Quantum Neural Networks (QNN) and Variational Quantum Circuits (VQC) which makes calculations more efficient and more reasonably anticipates predictions. How does QBrainNet solve this problem? QBrainNet uses quantum computing to process high dimension medical image data more efficiently and particularly, when the dimension of our data is under such small conditions as are illustrated in the conventional models (there are few distinct images in the background).

We first conduct a comprehensive evaluation where it is demonstrated that QBrainNet outperforms classical machine learning models (e.g., CNN, SVM, RF, KNN, and LR) in several critical metrics, i.e., accuracy, precision, recall, F1-score, AUC-ROC, and computational speed. We find that QBrainNet has a strong ability to identify strokes and little misclassifications precisely and performs better in different configurations of hyperparameters. For instance, our model obtains better AUC-ROC scores and shows merits with varying learning rates, adequately suggesting its flexibility and generalization capability on an extensive range of medical imaging data.

Furthermore, the Feature Importance Visualization highlights which features are the most important by prioritizing those for stroke detection. Thus, the model is better interpreted, and it provides some insight into the decision-making process. The Confusion Matrix depicts the application of a low false positive and false negative rate, among other things, supporting early stroke detection.

Although its training time is slightly higher than that of traditional models, QBrainNet is comparable in real-time prediction time, considering its similar inference time. QBrainNet is a promising tool for clinical applications that allows for real-time decision-making.

### 5.1 Future work

QBrainNet is a promising tool for predicting stroke; however, QBrainNet has some potential room for further development and enhancements. Second, the model can be corroborated in addition to the addition of more diverse and big medical imaging datasets, which could contain data from other imaging modalities (e.g., CT, MRI, ultrasound). The robustness of QBrainNet in real-world clinical scenarios and that the model behaves uniformly across various populations would need a large and diverse dataset for us to penetrate deeper.

It can also be optimized in the quantum components of QBrainNet both from the design point and from the quantum algorithmic perspective. With new and more efficient quantum algorithms emerging for these more than-ever powerful quantum computing technologies, new problems will arise. Further integrations of these advancements with the QBrainNet can lead to additional performance improvements, especially in speed and accuracy. Some of the tasks for exploring further are exploring the usage of more advanced quantum machine learning technologies such as quantum support vector machines or quantum k nearest neighbors that may help to improve data classification and pattern recognition.

Other than optimizing quantum components, QBrainNet could also be simplified to quantum-enhanced generative models. These models may generate medical images, mainly when insufficient data exists synthetically. We hypothesized that augmenting the dataset with high-quality synthetic quantum-enhanced images would allow us to train the model on a robust and more comprehensive dataset that would aid the model in generalization when processing unseen data.

Another important direction for future work is to explore the real-time deployment of QBrainNet in clinical settings. For this to be possible, the model would need to be integrated with the existing healthcare systems and its usage made practical for medical practitioners. Moreover, real-time performance evaluations and continuous learning mechanisms can be added to the model to enhance it with additional data as they become available. Integrating QBrainNet with electronic health records (EHR) and other clinical data sources can be a powerful tool for early stroke diagnosis to forecast timelines that can guide healthcare providers’ decisions.

Finally, investigating the explainability of QBrainNet for clinical decision-making is an integral part of future work. Although the model works very well, we need to understand how quantum-enhanced parts of the model can affect the predictions to gain the trust of healthcare providers. Since the decision-making in high-stakes applications, i.e., medical diagnostics, must be more transparent and interpretable, techniques such as model interpretability and explanation generation should be explored.

To summarize, QBrainNet is a very promising tool for using quantum enhancement to predict stroke, and further research and development in these areas are expected and necessary to advance its applicability in clinical use and ensure its success in the real world of healthcare.

## Data Availability

The original contributions presented in the study are included in the article/supplementary material, further inquiries can be directed to the corresponding authors.
